# Regeneration of actin filament branches from the same Arp2/3 complex

**DOI:** 10.1126/sciadv.adj7681

**Published:** 2024-01-26

**Authors:** Foad Ghasemi, LuYan Cao, Miroslav Mladenov, Bérengère Guichard, Michael Way, Antoine Jégou, Guillaume Romet-Lemonne

**Affiliations:** ^1^ Université Paris Cité, CNRS, Institut Jacques Monod, F-75013 Paris, France.; ^2^ The Francis Crick Institute, London, UK.; ^3^ Department of Infectious Disease, Imperial College, London, UK.

## Abstract

Branched actin filaments are found in many key cellular structures. Branches are nucleated by the Arp2/3 complex activated by nucleation-promoting factor (NPF) proteins and bound to the side of preexisting “mother” filaments. Over time, branches dissociate from their mother filament, leading to network reorganization and turnover, but this mechanism is less understood. Here, using microfluidics and purified proteins, we examined the dissociation of individual branches under controlled biochemical and mechanical conditions. We observe that the Arp2/3 complex remains bound to the mother filament after most debranching events, even when accelerated by force. Strikingly, this surviving Arp2/3 complex readily nucleates a new actin filament branch, without being activated anew by an NPF: It simply needs to exchange its nucleotide and bind an actin monomer. The protein glia maturation factor (GMF), which accelerates debranching, prevents branch renucleation. Our results suggest that actin filament renucleation can provide a self-repair mechanism, helping branched networks to sustain mechanical stress in cells over extended periods of time.

## Teaser

After dissociation of an actin filament branch, a new branch can grow from the same location using the same Arp2/3 complex.

## INTRODUCTION

In cells, branched actin filaments are involved in a variety of essential processes. They are key components of the cell cortex, of the lamellipodium at the front of a migrating cell, and of endocytic cups (*1*, *2*). Branched actin filaments are also important to displace and reshape mitochondria in the cytoplasm (*3*) and to repair damaged chromatin in the nucleus (*4*). These filament networks have specific dynamics, as both their assembly and disassembly are precisely controlled. In most of these processes, branched actin networks play a mechanical role, exerting forces as the filaments elongate, and adapting to mechanical loads (*5*–*7*).

An actin filament branch is nucleated by the Arp2/3 complex bound to the side of a preexisting “mother” filament [reviewed in (*8*)]. The Arp2/3 complex is composed of seven subunits, including actin-related proteins (Arp) 2 and 3, which can template the polymerization of an actin filament. In its basal state, however, the Arp2/3 complex adopts an inactive conformation where Arp2 and Arp3 are splayed apart and adopt a twisted conformation similar to monomeric actin (G-actin), and thus cannot nucleate a filament (F-actin) (*9*–*13*). Activation is mediated by membrane-anchored nucleation-promoting factors (NPFs), whose Verprolin-Central-Acidic (VCA) domains simultaneously bind the Arp2/3 complex and G-actin. This results in a conformational change of the Arp2/3 complex, which allows it to bind to the side of the mother filament, likely causing further conformational changes that lead to its activation (*14*). Upon detachment of the VCA domains (*15*), a new barbed end will elongate from the Arp2/3 complex and form a branch, or “daughter” filament, with a well-characterized 70° angle with respect to the mother filament (*16*). In the Arp2/3 complex at the branch junction, Arp2 and Arp3 are in a filament-like conformation (*17*, *18*).

Like actin, Arp2 and Arp3 have a nucleotide-binding pocket. The nucleation of the branch requires the Arp2/3 complex to be loaded with adenosine triphosphate (ATP), which will be hydrolyzed after the branch has nucleated (*19*–*22*). As long as it remains in the branch junction, the Arp2/3 complex is not able to exchange its nucleotide, which will thus be a marker of its age: Older branches will have an adenosine diphosphate (ADP)–Arp2/3 complex at their junction.

Branches eventually dissociate from the mother filament, thereby contributing to the reorganization and the turnover of the branched network. Despite its importance for the control of actin turnover, the mechanism of branch dissociation is less understood than that of branch nucleation (*23*, *24*). In vitro studies have shown that ATP hydrolysis in the Arp2/3 complex favors branch dissociation: “Older” ADP-Arp2/3 branches dissociate more easily (*21*, *23*, *25*). Further, the Arp2/3 complex can be directly targeted by the protein glia maturation factor (GMF) to accelerate debranching, or by the protein cortactin to stabilize the branch (*25*–*30*). Actin-binding proteins of the ADF/cofilin family also favor branch dissociation (*31*, *32*). In addition, Pandit *et al.* (*25*) have recently shown that the application of sub-piconewton pulling forces to branches dramatically accelerates their dissociation. This observation is of prime importance because branched actin networks are exposed to mechanical forces in cells, and this raises the question of how they are able to sustain prolonged mechanical stress. It also means that one needs to control the mechanical context to properly study branch dissociation.

Despite these recent advances, basic aspects of branch dissociation are still unclear. For instance, whether the Arp2/3 complex remains on the mother filament or departs with the dissociated branch remains elusive (*33*). Nonetheless, it is often considered that branch dissociation results from the detachment of the Arp2/3 complex from the mother filament (*16*, *25*). In this scenario, the pointed end of the dissociated branch could remain capped by the Arp2/3 complex, preventing depolymerization and reannealing with a free barbed end. Recent cryo–electron microscopy (EM) data of branch junctions provide accurate descriptions of buried surface areas between the Arp2/3 complex and the mother and daughter filaments (*17*, *18*, *34*). Yet, buried surface areas only provide a rough indication of the free energy associated with these interfaces (*35*), and their relative strengths remains an open question.

To address this question, we have monitored the dissociation of branches in controlled mechanical conditions. We find that not only does the Arp2/3 complex remain bound to the mother filament after nearly all debranching events but also, strikingly, it can then rapidly reload fresh ATP and nucleate a new branch, without being reactivated by NPFs.

## RESULTS

All experiments were carried out in vitro at 25°C, using purified proteins (from mammalian origin unless specified otherwise), in a buffer at pH 7.0 containing 50 mM KCl (see Materials and Methods). In most experiments, we used α-skeletal actin, but we also used γ-cytoplasmic actin where specified.

### Branch dissociation and renucleation

**Fig. 1.**
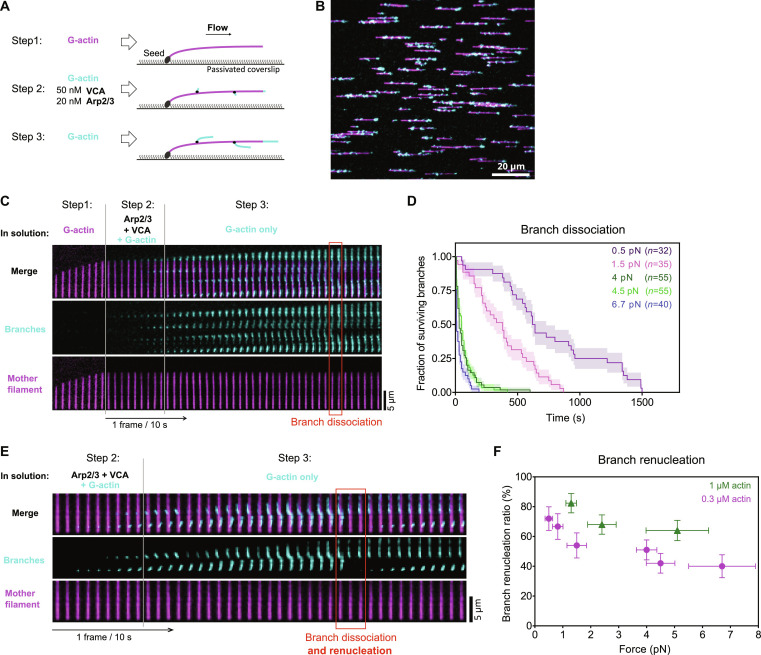
New actin branches can grow from the same Arp2/3 complex after a branch dissociation event. (**A**) Schematic of a typical branching and debranching experiment using microfluidics. Filaments are first elongated from surface-anchored spectrin-actin seeds by flowing in Alexa Fluor 488 (10%)–G-actin (magenta). Branches are then nucleated by flowing in Alexa Fluor 568 (10%)–G-actin (cyan) with VCA and Arp2/3. They are further elongated by flowing in Alexa Fluor 568 (10%)–G-actin alone. The elongation and dissociation of branches can then be monitored over time. The flowing solution applies a pulling force to the mother filament and the branches. (**B**) Portion of a TIRF microscopy field of view, showing mother filaments (magenta) and branches (cyan) formed as indicated in (A). (**C**) Time lapse showing the elongation of a mother filament (magenta), the nucleation of branches (cyan), their elongation, and the dissociation of a branch (red box). (**D**) Pulling forces accelerate the dissociation of branches. Each curve shows the surviving fraction of *n* branches monitored over time for a different experiment [branches aged for 20 min with 0.3 μM actin, between steps 2 and 3 indicated in (A)]. Each experiment was carried out with a different flow rate, to apply different forces, indicated as the average force applied to the branches as they dissociated. (**E**) Time lapse, similar to the one in (C), showing an example of branch renucleation (red box). (**F**) Pulling forces moderately reduce the fraction of branches that renucleate after dissociation. Each point is from a single experiment, analyzing, from left to right, *n* = 32, 30, 35, 55, 55, and 40 branches (0.3 μM actin) and *n* = 34, 50, and 50 branches (1 μM actin). Error bars are SDs.

We used a microfluidics assay (*36*–*38*) to monitor the nucleation and the dissociation of branches while exposing them to controlled mechanical tension (Fig. 1A). We first elongated filaments from surface-anchored seeds by flowing fluorescently labeled actin monomers (G-actin) in the microchamber. We subsequently generated branches by flowing in the Arp2/3 complex together with the VCA region of the NPF N-WASP and G-actin labeled with a different fluorescent color to facilitate the identification of branches, which tend to align with the mother filament because of the flow (see Materials and Methods). We next flowed labeled G-actin alone in the microchamber to monitor the elongation and the dissociation of the branches without nucleating new branches. This allowed us to keep the branch density low enough to clearly monitor the fate of individual branches (Fig. 1, B and C).

We quantified the dissociation of branches over time, as we exposed them to different flow velocities and thus to different forces [see Materials and Methods and (*37*, *38*)]. Similar to Pandit *et al.* (*25*), we observed that branches dissociated faster from mother filaments when exposed to increasing forces in the piconewton range (Fig. 1D).

Unexpectedly, however, we observed that the majority of dissociation events were followed by the growth of a new branch from the same location (Fig. 1E and fig. S1). The regeneration of the branches took place immediately after branch dissociation, with no detectable lag within the resolution of our experiments (fig. S2). The fraction of branches that regenerated after dissociating, a percentage we call the “branch renucleation ratio,” decreased with tension, but remained above 50% for forces up to 4 pN (Fig. 1F). We also observed that the branch renucleation fraction increased with G-actin concentration (Fig. 1F). The branch renucleation events took place in the absence of the Arp2/3 complex and VCA in solution, implying that the Arp2/3 complex remained on the mother filament upon dissociation of the branch, and that a new branch could be nucleated from that same Arp2/3 complex.

Actin filaments rarely fragment in our assay, in the absence of specific severing proteins such as ADF/cofilin (*38*, *39*). Nevertheless, any severing event occurring close to the branching junction would be mistaken for a dissociation event, and the subsequent elongation of the severed branch would be wrongly interpreted as renucleation. We thus sought to determine whether our observation of branch dissociation events was contaminated by branch severing events. First, we verified that the branch renucleation ratio was unaffected by changes in actin fluorescent labeling and illumination (fig. S3A). Second, we designed a specific experiment to test if any actin subunits from the first nucleation event were still present in the renucleated branch (fig. S3, B to D). This experiment is similar to Fig. 1A, except that we used G-actin with a high labeling fraction of a third fluorophore for the branching reaction (step 2), resulting in branches with a clearly labeled pointed end. After branch dissociation and the nucleation of new branches, these fluorescently labeled actin subunits could no longer be detected at the branch junction, in illumination conditions where single fluorophores could be detected. We conclude that the branch departure events that we observe are genuine branch dissociation events, where no actin subunits from the initial daughter filament remain bound to the Arp2/3 complex associated to the mother filament.

Our observation that the Arp2/3 complex remained bound to the mother filament upon dissociation of the branch appeared to contradict Pandit *et al.* (*25*) who detected no fluorescently labeled Arp2/3 complex on the mother immediately after branch dissociation. We wondered if this difference could come from our using mammalian Arp2/3 complex and mammalian actin, while Pandit *et al.* used the Arp2/3 complex from fission yeast and mammalian actin. To test this hypothesis, we repeated our experiments with the Arp2/3 complex from budding yeast and observed a branch renucleation ratio of less than 10% (fig. S4B). Our interpretation is that the interface between the mother filament and the Arp2/3 complex is much weaker when the latter is from yeast, while the former is made of mammalian actin. Consistently, we find that branches dissociate much faster when using yeast Arp2/3 complex instead of mammalian Arp2/3 complex (fig. S4A).

### Impact of force orientation

**Fig. 2.**
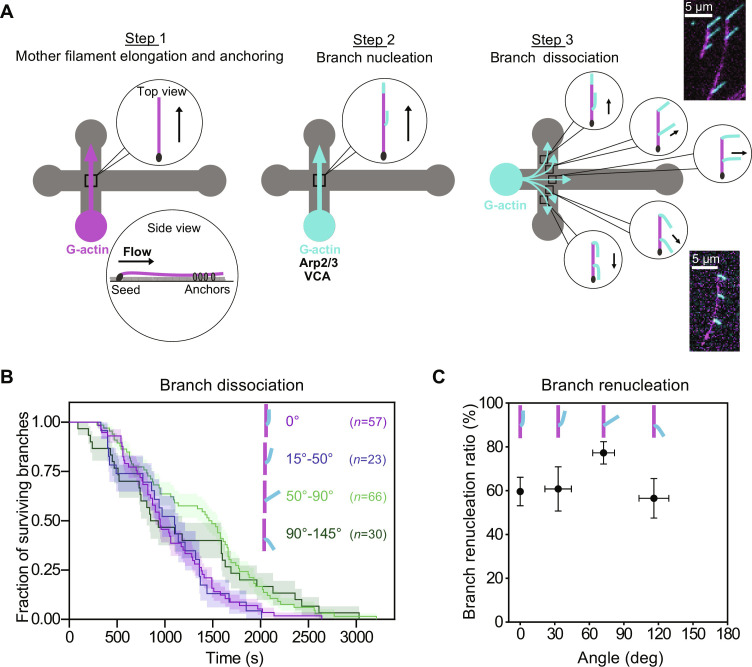
Impact of force orientation on debranching and renucleation. (**A**) Schematic of the assay where branches are pulled on with different orientations. Mother filaments (magenta) are elongated with a biotinylated segment, which is anchored to the surface (step 1). During the debranching and renucleation phase (step 3), the pulling force follows the direction of the flow, which makes different angles with respect to the mother filaments in different regions of the microfluidics chamber. The microscope images show examples from two different fields, with forces applied on branches at 30° to 47° (top image) and at 114° to 120° (bottom image) with respect to their mother filaments. (**B**) Each curve shows the surviving fraction of branches monitored over time for a different population of *n* branches from nine independent experiments pooled together according to the angle between the applied force and the mother filament. (**C**) The direction of the pulling force has a moderate impact on the branch renucleation ratio, computed for each of the four populations of branches whose detachment is shown in (B). The value for 50° to 90° is significantly higher than for smaller angles (*P* = 0.033, two-sided Fisher’s exact test). Error bars indicate binomial SDs.

In our standard experiment, the mother filaments aligned with the flow, and thus, the pulling force applied to the branches is parallel to the mother filaments (Fig. 1). We wondered if the orientation of the force affects the branch dissociation rate and renucleation. To vary the angle between the mother filament and the flow, we modified our assay as follows (Fig. 2 and Materials and Methods). We worked in the region of the microchamber where the channels intersect, so we can orient the flow of the incoming solutions in different directions. We grew filaments from surface-anchored seeds by flowing in fluorescently labeled G-actin, as before, but we then elongated them further with a solution supplemented with biotinylated G-actin. This created a biotinylated filament segment, which we could then anchor to surface-anchored biotin-BSA, by flowing in neutravidin. As a result, the orientation of mother filaments was fixed, imposed by the anchored seed and the anchored biotinylated segment, regardless of the orientation of the flow. We then nucleated and elongated branches, and monitored their fate as before. To avoid potential artifacts due to local anchoring points, we only followed branches whose junction was in the unanchored segment of the mother filament.

By flowing the G-actin solution from one channel into the three others, we obtained different local flow directions so that applied forces have different angles with respect to the mother filaments, within the same microchamber (Fig. 2A). Mother filaments whose biotinylated segment failed to bind the surface provided local control situations where the mother and the branch were aligned with the flow. In a separate experiment, we verified that the local flow velocity was the same for the different flow directions by tracking the movement of micrometer-size beads (fig. S5). The forces we applied to the actin filament branches thus had different orientations but similar amplitudes (approximately 1 pN).

We pooled branches into four subpopulations, based on the angle Θ between the local direction of the flow (i.e., the direction of the applied force) and the mother filament. We found that branches dissociated at a similar rate from their mother filaments, regardless of the orientation of the force (Fig. 2B). This is reminiscent of recent observations by Pandit *et al.* (*25*) using fission yeast Arp2/3 complex and mammalian actin. We found that the branch renucleation ratio was very mildly affected across the range of angles we studied (0 to 145°). The branch renucleation ratio was approximately 30% higher when Θ was in the vicinity of 70°, the canonical angle of the branch junction (Fig. 2C).

These results show that branch renucleation following debranching is a general mechanism, taking place regardless of the orientation of the pulling force. The higher branch renucleation ratio measured around Θ = 70° indicates that the Arp2/3 complex is even more likely to remain bound to the mother filament when the tension is applied without bending the branch and without shearing the branch junction.

### Actin isoform and profilin

**Fig. 3.**
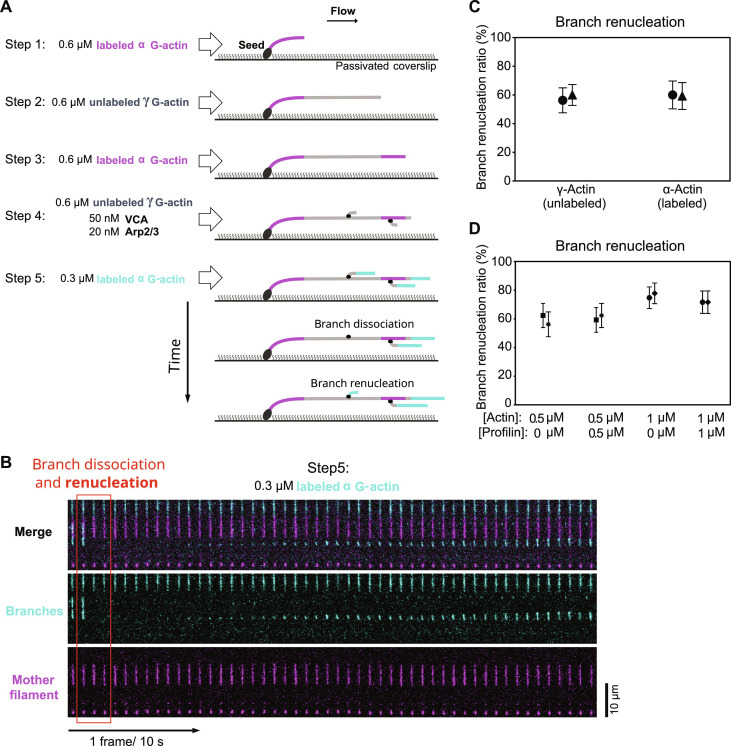
Branch renucleation is not actin isoform specific and occurs in the presence of profilin. (**A**) Schematic of experiment using microfluidics to study branch junctions made with unlabeled cytoplasmic γ-actin. The mother filaments comprise a long segment made with unlabeled cytoplasmic γ-actin (steps 1 to 3), and the branches are nucleated using unlabeled cytoplasmic γ-actin (step 4). (**B**) Time lapse showing a branch dissociating and renucleating, from the unlabeled segment of the mother filament, during step 5 of the experiment described in (A). (**C**) The branch renucleation ratio is similar for mother filaments made with unlabeled cytoplasmic γ-actin or with Alexa Fluor 488–labeled (10%) α-skeletal actin. (**D**) The branch renucleation ratio is not affected by adding equimolar amounts of profilin to G-actin during the final step of the experiment (branch dissociation and renucleation, step 3 in Fig. 1A). In (C) and (D), each data point is from one experiment, monitoring *n* = 32, 45, 25, and 27 dissociating branches (from left to right) for experiments in (C) and *n* = 32 dissociating branches for each experiment in (D). Points with matching symbols are from observations carried out simultaneously, in different regions of the same microfluidics chamber. Error bars indicate SDs.

Our results so far imply that, following the majority of branch dissociation events, the Arp2/3 complex remains on the mother filament and nucleates a new branch. Since we made these observations using α-skeletal actin, we wondered whether they would differ if we used cytoplasmic actin. To be closer to physiological conditions, where actin is unlabeled, we modified our assay to monitor branch junctions made with unlabeled cytoplasmic γ-actin (Fig. 3, A and B, and Materials and Methods). Briefly, we successively flowed different actin solutions in the microchamber to elongate mother filaments comprising a long segment made entirely of unlabeled γ-actin, between two segments made of labeled α-actin from skeletal muscle. Next, we nucleated branches using unlabeled γ-actin. Finally, we elongated the branches and monitored their outcome while continuously flowing labeled α-actin in the chamber. This enabled us to monitor the dissociation of branches where only unlabeled γ-actin was in contact with the Arp2/3 complex, in both the mother filament and the pointed region of the branch. We found that the branch renucleation ratio was the same as for our standard branches, monitored simultaneously in the same microchamber (Fig. 3C and Materials and Methods).

We also wondered if our results could be specific to the NPF we used to nucleate the first branches, VCA from N-WASP. We thus repeated our experiments using VCA from WASP and found that 55% (±11%, *n* = 22) of branches were regenerated after dissociating from the mother filament. This is similar to what we observed for branches nucleated using VCA from N-WASP.

Since, in cells, available actin monomers are mostly in complex with profilin (*40*, *41*), we asked whether profilin could affect branch renucleation. We compared, side-by-side in the same microchamber, the behavior of branches in the presence of actin alone and supplemented with profilin (Fig. 3D). We found that adding equimolar amounts of profilin to actin had no impact on the branch renucleation ratio.

### Nucleotide exchange on surviving docked Arp2/3 complex

Having established that branch regeneration is a robust mechanism, we next sought to better understand the underlying molecular mechanism. Earlier reports indicate that the Arp2/3 complex needs to be loaded with ATP to be activated by an NPF and nucleate a branch (*19*, *20*). This led us to wonder whether this was also the case for the renucleation events we observed. To determine if branches were renucleated from ADP- or ATP–Arp2/3 complex, we took advantage of the fact that ADP-Arp2/3 branches dissociate faster (*21*, *23*, *25*).

**Fig. 4.**
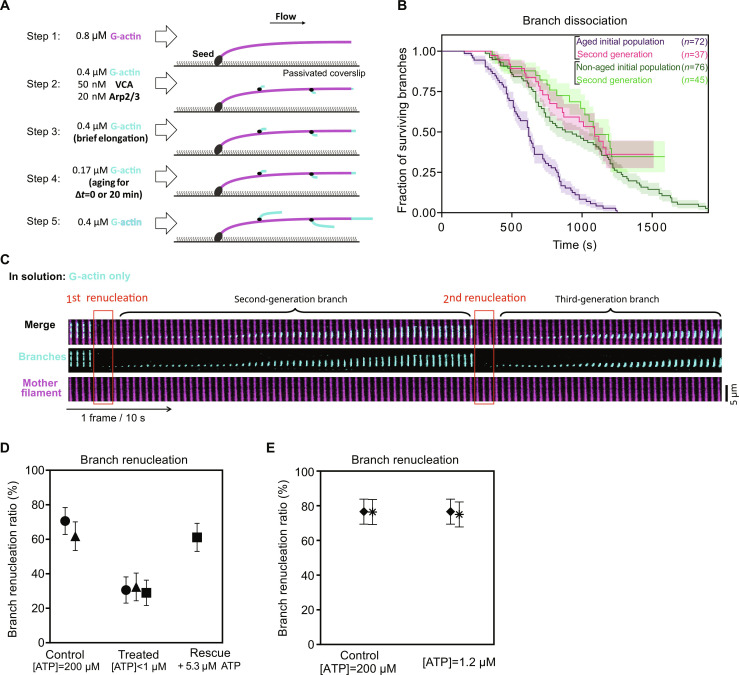
Arp2/3 complex binds fresh ATP to renucleate a branch. (**A**) Schematic of the aging experiment using microfluidics. Compared to the standard assay shown in Fig. 1, an extra step (step 4) is added to age the branch junctions without elongating the branches: A lower concentration of G-actin is flowed in for 0 min (non-aged branches) or 20 min (aged branches). (**B**) Kinetics of branch dissociation, for aged (purple curve) and non-aged branches (dark green curve), and for the branches that have renucleated in each case (“second generation”). (**C**) Time lapse showing a branch detaching and renucleating, twice. In this experiment, 71% of the initial branches (*n* = 41) renucleated and 69% of the second generation of branches (*n* = 29) renucleated. (**D**) The branch renucleation ratio decreases when ATP is depleted from the buffer, and is rescued by adding back ATP. (**E**) The branch renucleation ratio is not affected when ATP is diluted to 1.2 μM. In (D) and (E), each data point is from one experiment, monitoring (from left to right) *n* = 34, 34, 36, 34, 38, and 36 dissociating branches for experiments in (D) and *n* = 34, 30, 35, and 30 dissociating branches for each experiment in (E). Points with matching symbols are from observations carried out simultaneously, in different regions of the same microfluidics chamber. Error bars indicate SDs.

To obtain ADP-Arp2/3 branches, we aged them for 20 min before monitoring their dissociation (Fig. 4A). As expected, in our experiments, the surviving fraction of aged branches (i.e., mostly ADP–Arp2/3 complex at the start of the experiment) decreased faster than that of non-aged branches (i.e., ATP–Arp2/3 complex and ADP-Pi–Arp2/3 complex at the start of the experiment) (Fig. 4, A and B). Strikingly, in both situations, we observed that the renucleated branches dissociated slowly, like non-aged branches (Fig. 4B). This suggests that the Arp2/3 complex is loaded with ATP when it renucleates a filament, after dissociation of the first branch.

The fact that aged branches dissociate faster than non-aged branches (Fig. 4B) implies that most surviving docked Arp2/3 complexes are in the ADP state. Thus, our result that branches are renucleated from the ATP–Arp2/3 complex indicates that the Arp2/3 complex exchanges its nucleotide after branch dissociation to nucleate a new branch. Consistently with this, we observed that renucleated branches, after they dissociated, were renucleated again, with the same branch renucleation ratio (Fig. 4C). This could be observed for multiple generations, as if each renucleation event was independent of the past history of that specific Arp2/3 complex.

To further confirm the notion that the Arp2/3 complex remaining on the mother filament exchanges its nucleotide to nucleate a new branch, we decreased the ATP concentration to see if that would impair branch renucleation. To maintain G-actin in the ATP state, we did not fully remove ATP from solution. We verified, by measuring the elongation rate at filament barbed ends, that all the G-actin was indeed loaded with ATP in our experiments, even at our lowest concentrations of ATP. We observed that branches dissociating while exposed to G-actin in an ATP-deficient solution ([ATP] < 1 μM, see Materials and Methods) renucleated less than half as frequently as in our control experiment ([ATP] = 200 μM) carried out simultaneously in the same microchamber (Fig. 4D). This result confirms that ATP in solution is required for branch renucleation. We were not able to accurately measure ATP concentrations below 1 μM, and thus could not determine its affinity for the Arp2/3 complex bound to the mother filament. However, we measured that branch renucleation was unaffected when the ATP concentration was decreased as low as 1.2 μM (Fig. 4E).

Together, our results indicate that actin filament branches dissociate from the ADP–Arp2/3 complex, which remains on the mother filament, and that this surviving docked Arp2/3 complex is able to rapidly exchange its nucleotide to become ATP–Arp2/3 complex and renucleate a branch.

### Detachment of surviving docked Arp2/3 complex

To estimate the rate at which the surviving docked Arp2/3 complex exchanges its nucleotide, we needed to take into account competing reactions. We reasoned that, following branch dissociation, the surviving docked ADP–Arp2/3 complex can either exchange its nucleotide to become ATP–Arp2/3 complex or dissociate from the mother filament. In turn, the ATP–Arp2/3 complex can either dissociate from the mother filament, or bind an actin monomer, which we assumed would stabilize its interaction with the mother filament as in a new branch junction. This last assumption is consistent with our observation that the branch renucleation ratio increases with the G-actin concentration (Fig. 1F).

**Fig. 5.**
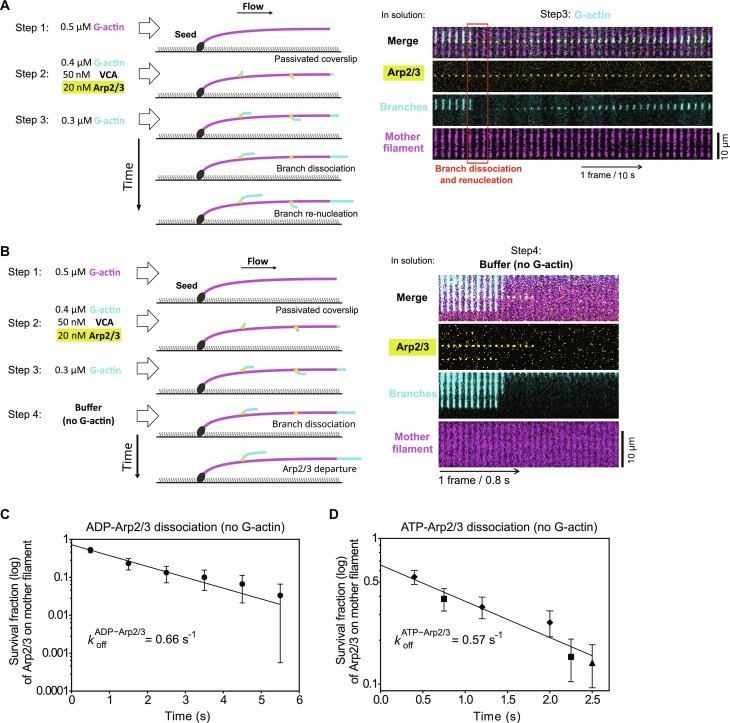
Dissociation of Arp2/3 complex from the mother filament following debranching. (**A**) Schematic and time lapse of an experiment carried out with the Alexa Fluor 488–labeled Arp2/3 complex, showing branch dissociation and renucleation. Similar to experiments with the same conditions and unlabeled Arp2/3 complex, 70% of the dissociated branches (*n* = 23) were renucleated. (**B**) Schematic and time lapse of an experiment carried out with the fluorescently labeled Arp2/3 complex in the absence of G-actin during the final step (branch dissociation). Note the shorter time interval between images in this experiment, to monitor the departure of the Arp2/3 complex from the mother filament, following the dissociation of the branch. (**C**) Fraction of Arp2/3 complex bound to the mother filament (log scale) as a function of time, observed as described in (B), using an ATP-free buffer in step 4. The data are pooled from four experiments (*n* = 6, 15, 7, and 2 branches). (**D**) Fraction of Arp2/3 complex bound to the mother filament (log scale) as a function of time, observed as described in (B), using our standard buffer with 200 μM ATP in step 4. The data are pooled from different experiments using different time intervals: 0.8 s (diamonds, nine experiments with *n* = 4, 5, 8, 7, 4, 4, 14, 10, and 2 branches); 1.5 s (squares, seven experiments with *n* = 14, 14, 4, 3, 7, 1, and 9 branches); 5 s (triangle, two experiments with *n* = 26 and 31). In (C) and (D), time *t* = 0 corresponds to the dissociation of the branch. In these experiments [(B) and (C)], branches were exposed to a pulling force between 2.2 and 2.9 pN.

We thus sought to quantify the dissociation of the Arp2/3 complex from the mother filament. To do so, we fluorescently labeled the Arp2/3 complex (see Materials and Methods) and verified that this labeling did not affect the nucleation, dissociation, and renucleation of the branches (Fig. 5A). We could thus directly visualize the same Arp2/3 complex remaining bound to the mother filament throughout branch dissociation and renucleation.

We then performed experiments in the absence of G-actin (buffer only) during the debranching step, to monitor the dissociation of the Arp2/3 complex from the mother filament following the dissociation of the branch (Fig. 5B). Experiments were performed in an ATP-free buffer to measure the off-rate of ADP–Arp2/3 complex (Fig. 5C), as well as in our standard buffer (200 μM ATP) to measure the off-rate of the ATP–Arp2/3 complex (Fig. 5D). We found similar dissociation rates for both situations: *k*_off_ = 0.66 (±0.16) s^−1^ for the ADP–Arp2/3 complex (Fig. 5C) and 0.57 (±0.22) s^−1^ for the ATP–Arp2/3 complex (Fig. 5D). Knowing the off-rate for the ADP–Arp2/3 complex, and considering that the branch renucleation ratio is within 5% of the plateau value with 1.2 μM ATP (Fig. 4E, based on our error bars), we can determine that the effective rate constant for ATP reloading is greater than 6 μM^−1^ s^−1^. This ensures that, with 200 μM ATP, nucleotide exchange is orders of magnitude faster than the dissociation of the ADP–Arp2/3 complex, and that it is indeed the dissociation of the ATP–Arp2/3 complex that is observed.

In addition, the exponential fits of the survival curves of Arp2/3 complexes on the mother filament (Fig. 5, C and D) allow us to estimate (*y* intercepts) that approximately 70% of Arp2/3 complexes remained on the mother filament upon debranching, in these experiments where we applied a 2.2- to 2.9-pN pulling force to the branches (to accelerate debranching and minimize the photobleaching of the Arp2/3 complex while we acquired images at a rate of 0.2 to 1.25 Hz, see Materials and Methods). Our estimation of 70% Arp2/3 complexes remaining on the mother filament is consistent with the branch renucleation ratios we have measured in this force range (Fig. 1F).

### Quantitative model for branch dissociation and renucleation

**Fig. 6.**
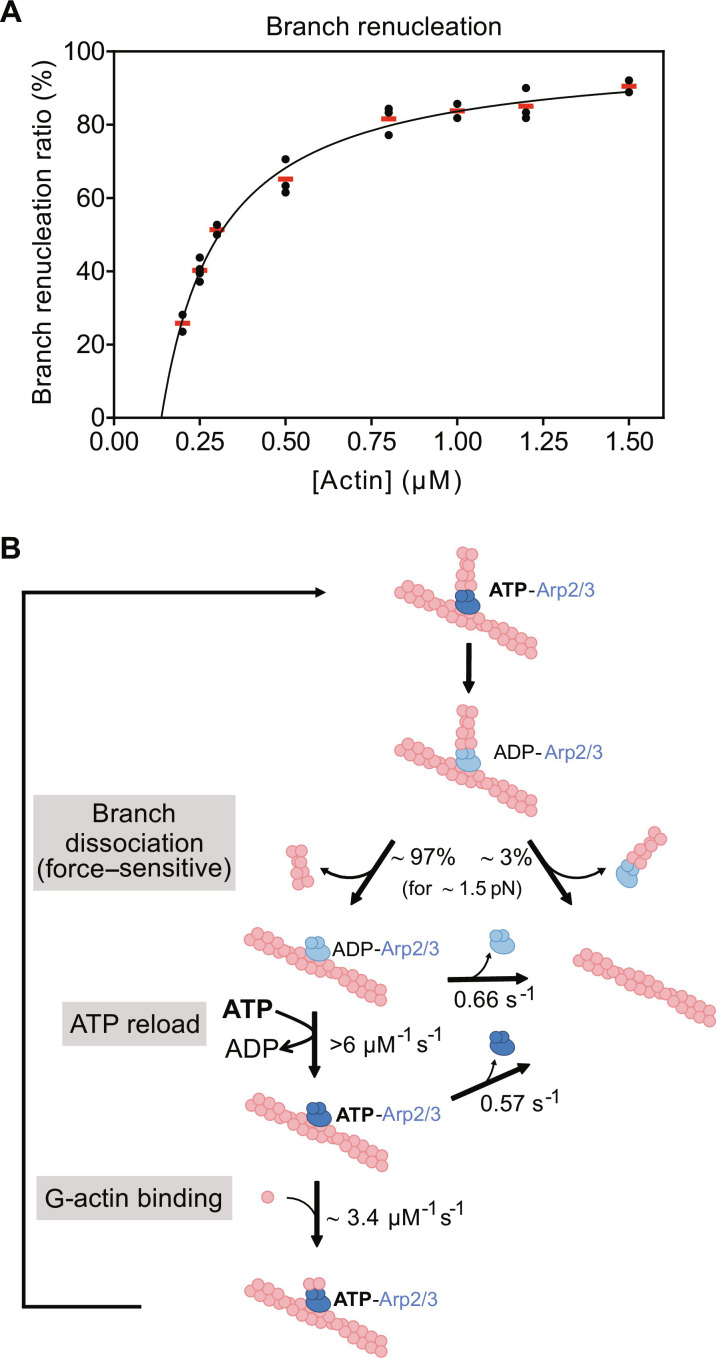
Impact of G-actin concentration on branch renucleation and model for debranching and branch renucleation. (**A**) Impact of G-actin concentration on the branch renucleation ratio. Each black data point is from a single experiment, monitoring *n* dissociating branches: *n* = 17, 32 (0.2 μM actin); 35, 32, 38, 32 (0.25 μM); 32, 36 (0.3 μM); 34, 30, 26 (0.5 μM); 32, 36, 35 (0.8 μM); 14, 22 (1 μM); 22, 30, 20 (1.2 μM); 36, 38 (1.5 μM). Red bars indicate average values for each actin concentration. Branches were exposed to a pulling force of 1.54 (±0.23) pN. The solid line is a fit, considering that renucleation occurs when an actin subunit is added to the Arp2/3 complex before it detaches from the mother filament (see Materials and Methods). (**B**) Global model for debranching and branch regrowth. Aged actin filament branches dissociate in a force-sensitive manner, most often leaving an ADP–Arp2/3 complex bound to the mother filament. This surviving Arp2/3 complex will nucleate a new branch, provided that it can load fresh ATP and bind G-actin before detaching from the mother filament.

In our description of the different pathways leading either to the dissociation of the Arp2/3 complex from the mother filaments or to the renucleation of a branch, we hypothesized that the binding of an actin monomer to the ATP–Arp2/3 complex would stabilize its interaction with the mother filament and promote the nucleation of a new branch. This is consistent with our observation that the branch renucleation ratio increases with the G-actin concentration (Fig. 1F). To quantify this reaction, we measured the branch renucleation ratio over a broader range of G-actin concentrations (Fig. 6A). We found that it follows a typical saturation curve, with less than 25% of branches renucleating below 0.2 μM G-actin, and more than 85% of branches renucleating above 1.5 μM. Knowing the rate constant of the competing reaction (dissociation of the ATP–Arp2/3 complex from the mother filament; Fig. 5D), we can fit this curve to estimate that G-actin binds to the mother-bound ATP–Arp2/3 complex with rate constant *k*_on_ = 3.4 (±1.24) μM^−1^ s^−1^ and a critical concentration *C*_c_ = 0.14 (±0.2) μM. The fit also yields a plateau value of 97(±3)%, which is an estimation of the fraction of debranching events that leave the Arp2/3 complex bound to the mother filament, in the conditions of this experiment where a 1.54 (±0.23) pN force was applied to the branches.

With these last measurements, we have quantified the final step of the global reaction scheme leading to branch renucleation (Fig. 6B). This scheme can be summarized as follows. Over time, the Arp2/3 complex in the branch junction hydrolyzes its ATP, and debranching then becomes more likely. This branch dissociation step is accelerated by mechanical forces, which also favor the departure of the Arp2/3 complex from the mother filament. Nonetheless, up to several piconewtons of applied force, the vast majority of debranching events occur with the Arp2/3 complex remaining bound to the mother filament. This ADP–Arp2/3 complex remaining on the mother filament then rapidly exchanges its nucleotide, reloading fresh ATP from solution. If [ATP] > 1 μM, this reaction nearly always occurs before the dissociation of the ADP–Arp2/3 complex from the mother filament. The mother-bound ATP–Arp2/3 complex can then bind G-actin from solution, thereby nucleating a new branch that is identical to the initial branch. If the concentration of G-actin is greater than a few micromolars, this reaction nearly always occurs before the dissociation of the ATP–Arp2/3 complex from the mother filament. These rapid reaction rates explain why branches appear to renucleate instantly, within the resolution of our experiments (fig. S2).

### Impact of GMF

**Fig. 7.**
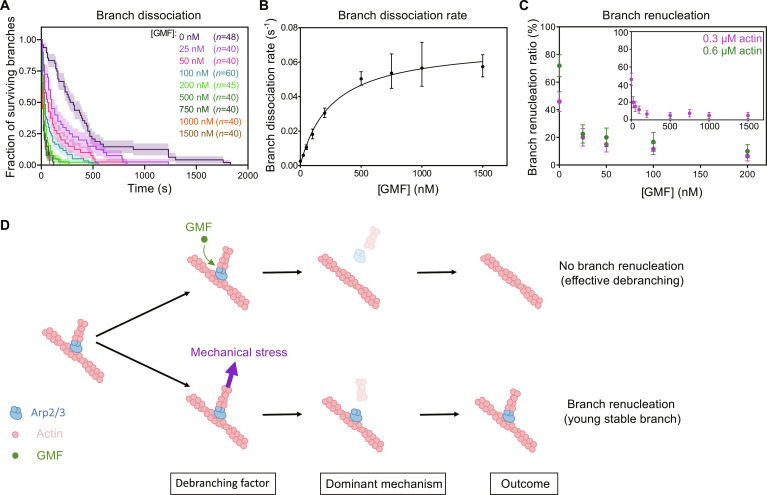
Impact of GMF on branch dissociation and branch renucleation. (**A**) Fraction of remaining branches over time, for different GMF concentrations (after 30 min of aging, and with an average 1-pN pulling force applied to the branches). (**B**) Branch dissociation rate versus GMF concentration. The rates were determined by exponential fits of the survival curves shown in (A), and the error bars are asymmetrical 95% confidence intervals (see Materials and Methods). The solid line is a fit by a Michaelis-Menten equation, assuming a rapid equilibrium between GMF and the branch junction. The fit yields a dissociation constant *K*_D_ = 260 (±117) nM, a maximum debranching rate *k*_max_ = 0.071 (±0.008) s^−1^, and a debranching rate in the absence of GMF comprised between 0 and 0.005 s^−1^ (95% confidence interval). (**C**) Branch renucleation ratio as a function of GMF concentration for two different actin concentrations. The data at 0.3 μM actin are from the same dataset as in (A). The data points at 0.6 μM actin each represent an independent experiment, monitoring *n* = 32, 40, 35, 30, and 40 dissociating branches, from left to right. Inset: Data at 0.3 μM actin shown over a larger range of GMF concentrations. (**D**) Schematic summarizing our interpretation of the different outcomes based on which factor promotes debranching. If branch dissociation is caused by GMF, the dominant outcome is an effective debranching, resulting in a linear filament with no branch (we propose that GMF first dissociates the branch from the Arp2/3 complex, and then dissociates the surviving Arp2/3 complex from the mother filament). In contrast, if debranching is a consequence of mechanical stress, the dominant outcome is the dissociation of the branch followed by the rapid nucleation of a new branch by the same ATP-reloaded Arp2/3 complex. This results in a reinforcement of the branch junction.

We wondered whether the global reaction scheme leading to branch renucleation (Fig. 6B) could be affected by regulatory proteins. To address this question, we monitored branch dissociation and renucleation in the presence of glia maturation factor (GMF), a protein known to accelerate debranching (*26*). We used GMF from *Drosophila*, which is very similar to human GMF (fig. S6). We confirmed that GMF accelerates debranching, and we measured that it does so with an apparent dissociation constant *K*_D_ = 260 (±117) nM and a maximum debranching rate *k*_max_ = 0.071 (±0.008) s^−1^ (for the ADP–Arp2/3 complex and an average pulling force of 1 pN; Fig. 7, A and B). We also found that GMF drastically hinders branch renucleation (Fig. 7C). GMF seemed particularly efficient at preventing branch renucleation: In the presence of 25 nM GMF, where we can estimate that [GMF]/([GMF] + *K*_D_) = 9% of branch dissociation events are caused by GMF, the fraction of branches that renucleate in the presence of 0.6 μM actin was reduced more than threefold (Fig. 7C). This suggests that GMF can accelerate the dissociation of the surviving docked Arp2/3 complex from the mother filament, independently of its promotion of branch dissociation. We verified that GMF did not affect barbed end elongation in our experiments, confirming that GMF does not interact with actin, and indicating that GMF directly binds to the Arp2/3 complex to prevent branch renucleation.

Since GMF is expected to target the interface of the daughter filament with the Arp2/3 complex (*27*, *42*), we wondered if the Arp2/3 complex was left on the mother filament following GMF-mediated branch dissociation. We thus monitored the presence of fluorescently labeled Arp2/3 complex, with a 1-s time interval, as branches dissociated in the absence of G-actin and in the presence of 1 μM GMF (similar to Fig. 5C). We observed that, out of 20 Arp2/3 complexes that were visible at the branch junction, 6 were still visible on the first image where the branch was gone (i.e., 0.5 s on average after branch dissociation), and none were visible on the next image. Since 1 μM GMF is nearly saturating the acceleration of branch dissociation (Fig. 7B), nearly all debranching events were mediated by GMF. Our data are thus compatible with a scenario where GMF leaves the Arp2/3 complex on the mother filament during the debranching reaction, and subsequently accelerates its dissociation from the mother filament. However, our time resolution is not sufficient to determine the proportion of Arp2/3 complexes removed from the mother filament during, or after, the dissociation of the branch.

The impact of GMF on branch renucleation contrasts with that of pulling forces, which have a strong effect on the rate of branch dissociation and a moderate impact on branch renucleation (Fig. 1). It thus appears that the final outcome following a debranching event depends on what factor triggered debranching (Fig. 7D). When a branch dissociates because of mechanical stress, the Arp2/3 complex will most likely remain bound to the mother filament and nucleate a new branch, and this new branch will be more resistant because it has an ATP–Arp2/3 complex at its junction. In contrast, when branch dissociation is caused by GMF, it is likely that no branch will regrow.

## DISCUSSION

We show here that, in contrast with what is often assumed, the mammalian Arp2/3 complex stays bound to the mother actin filament when the branch dissociates. Further, the Arp2/3 complex that remains on the mother filament can readily nucleate a new branch, from the same location, without requiring reactivation by an NPF. We show that this is a robust mechanism, taking place over a wide range of conditions (Figs. 2 and 3). Nonetheless, the likelihood of branch renucleation can be reduced by pulling on the branch junction, and by decreasing the concentration of ATP or G-actin. This reflects the three requirements for branch renucleation (Fig. 6B): First, the Arp2/3 complex must stay on the mother filament upon debranching (slightly less likely when pulling on the branch); second, it must then exchange its nucleotide before detaching (less likely at very low ATP concentration); third, the ATP–Arp2/3 complex must bind actin monomers before detaching (less likely at very low G-actin concentration). The key for renucleation to occur is that ATP and actin bind the surviving Arp2/3 complex faster than the latter would dissociate from the mother filament, to stabilize it and nucleate a new branch. In contrast to these factors, which require extreme conditions (high forces, or nearly total depletion of ATP or G-actin) to affect branch renucleation, GMF at moderate concentration efficiently prevents the renucleation of dissociated branches (Fig. 7).

### Molecular insights

Quite strikingly, our data indicate that the dissociation of the branch does not inactivate the Arp2/3 complex remaining on the mother filament. During the initial activation of the Arp2/3 complex, binding to the mother filament can be viewed as the key final step, completing and stabilizing the conformational changes initiated by the binding of the NPF together with G-actin (*10*, *14*, *17*, *18*). Consistent with this view, our results indicate that, after the branch has dissociated, the mother–Arp2/3 complex interactions are able to maintain the Arp2/3 complex in a quasi-active conformation, where it only needs to reload ATP and bind actin to be again stabilized in its active conformation.

The presence of ATP in the Arp2/3 complex is required to nucleate the initial branch (*19*, *20*) and ATP hydrolysis promotes branch dissociation, which we show here to result predominantly from the rupture of the Arp2/3 complex–daughter interface. It thus seems that the nucleotide state of Arp2 and Arp3 is key for their interaction with actin subunits. In contrast, the nucleotide state of Arp2 and Arp3 does not seem to directly affect the interaction of the Arp2/3 complex with the mother filament. For instance, after debranching, loading ATP does not stabilize the interaction of the Arp2/3 complex with the mother filament (Fig. 5, C and D). Rather, it is the binding of actin to Arp2 and Arp3 that appears to stabilize the Arp2/3 complex in the conformation where it binds tightly to the mother filament (*14*, *18*). At this stage, it is unclear whether binding one actin monomer to Arp2 and Arp3 suffices to stabilize the Arp2/3 complex on the mother filament, or if more actin monomers are required.

In the lone Arp2/3 complex that remains on the mother filament when the branch dissociates, Arp2 and Arp3 exhibit a behavior that is reminiscent of both G-actin and F-actin. The rapid reloading of ATP (Fig. 4), similar to what has been reported for the free Arp2/3 complex (*19*, *20*), suggests that Arp2 and Arp3 should be in an open G-actin–like conformation, with accessible nucleotide-binding pockets. This seems to change once the Arp2/3 complex is loaded with ATP, as it can bind actin subunits with an on-rate that is only threefold lower than that of a filament barbed end (Fig. 6). If two actin subunits are required to stabilize the Arp2/3 complex on the mother filament, their on-rate constants would be approximately twofold higher than our estimation, and thus even closer to that of a filament barbed end. It thus appears that Arp2 and Arp3 are then in a conformation similar to that of a filament barbed end, where the two terminal actin subunits adopt an F-actin–like conformation, as recently shown by cryo-EM (*43*). At this stage, we do not know whether it is the nucleotide state of Arp2 or Arp3 that determines the behavior of the Arp2/3 complex during branch renucleation. Early reports suggest that ATP is hydrolyzed more rapidly in Arp2 (*19*), but recent cryo-EM data on the yeast Arp2/3 complex suggest that it could rather be in Arp3 (*18*).

Our quantification of the branch renucleation ratio provides decisive insights into the relative strength of the mother–Arp2/3 complex and Arp2/3 complex–daughter interfaces. Within the range of forces we have studied, the Arp2/3 complex remains bound to the mother filament during the vast majority of debranching events, showing that the mother–Arp2/3 complex interface is the stronger one. This is consistent with recent cryo-EM data indicating that cortactin stabilizes branch junctions by reinforcing the bond between the Arp2/3 complex and the branch (*30*). The acceleration of debranching with force indicates an overall “slip bond” behavior, where interactions are destabilized by force. The fact that force moderately affects the branch renucleation ratio indicates that both interfaces are similarly affected by force, and thus that they both behave like slip bonds. Yet, since the branch renucleation ratio decreases with force, it seems that the interface between the mother filament and Arp2/3 complex is slightly more sensitive to force than the Arp2/3 complex–daughter interface (see Materials and Methods on data analysis, for details). Note that, since the flow applies negligible forces to the surviving Arp2/3 complex and to the first actin subunits that bind to it (*37*), we expect that only the branch dissociation step can be force sensitive (Fig. 6B).

Our data also show that the Arp2/3 complex–daughter interface is weaker than the interface between actin subunits within the filament. This is consistent with our recent observations on linear (i.e., nonbranched) filaments nucleated by the Arp2/3 complex after activation by SPIN90 (*29*). In this situation, the need for a mother filament is bypassed and the Arp2/3 complex is bound simultaneously to SPIN90 and to the pointed end of the nucleated filament (*44*, *45*). We observed that the interface between the Arp2/3 complex and the filament ruptured first, leaving the Arp2/3 complex bound to SPIN90 with no actin subunits (*29*). This indicates that the interface between the SPIN90-activated Arp2/3 complex and the nucleated filament, which is very similar to the Arp2/3 complex–daughter interface in branch junctions (*17*), is weaker than the interface between actin subunits. The weakness of the Arp2/3 complex–daughter interface could stem from the specific conformation adopted by the D-loop of the first two actin subunits of the daughter filament in contact with Arp2 and Arp3, as recently observed in branch junctions using cryo-EM (*18*).

Our results showing that GMF prevents branch renucleation are reminiscent of our recent observations where GMF enhanced the detachment of the Arp2/3 complex from both SPIN90 and the pointed end of the filament (*29*). They also provide additional information on the interaction of GMF with the Arp2/3 complex. Cocrystal structures from GMF and ATP–Arp2/3 complex show that GMF binds primarily to Arp2 (*27*) and possibly also to Arp3 (*42*, *46*), and both of these interactions should destabilize the interface between the Arp2/3 complex and the daughter filament. Our data using the fluorescently labeled Arp2/3 complex are compatible with the notion that GMF dissociates the branch from the Arp2/3 complex, leaving the latter bound to the mother filament. Further, our data indicate that GMF is more efficient at preventing branch renucleation than at accelerating branch dissociation: The half-maximum effect is reached with less than 25 nM GMF for branch renucleation, and with 200 nM GMF for branch dissociation (Fig. 7). This suggests that GMF is able to promote the detachment of the Arp2/3 complex that remained on the mother filament after branch dissociation, independently of its ability to promote branch dissociation. These two reactions could possibly involve different binding sites. In our global reaction scheme (Fig. 6B), GMF could promote the dissociation of the surviving Arp2/3 complex by interfering with different steps. For example, GMF could bind Arp2 and hinder the reloading of fresh ATP, and/or the binding of actin monomers. Future studies should bring insights into the molecular mechanism(s) by which GMF prevents branch renucleation.

We also show that, while branch renucleation is the dominant outcome for branches made using the mammalian Arp2/3 complex and mammalian actin, it is rare if branches are made using the budding yeast Arp2/3 complex and mammalian actin (fig. S4B). Together with our observation that branches made with the yeast Arp2/3 complex and mammalian actin also dissociate faster (fig. S4A), this suggests that the interface between the mammalian actin mother filament and the yeast Arp2/3 complex is weaker than in an all-mammalian branch junction. Consistently, the fission yeast Arp2/3 complex was recently reported to dissociate from mammalian actin mother filaments upon debranching (*25*). This result possibly reflects a weaker interaction between proteins from evolutionarily distant species. Alternatively, it could reflect a specific difference between branch junctions in yeast and in mammals. Recent cryo-EM data highlight structural differences between the fission yeast and mammalian Arp2/3 complex at branch junctions, but so far, they report no major difference regarding the mother–Arp2/3 complex interface (*17*, *18*).

### Physiological implications

In animal cells, the cytoplasm typically contains 100 to 200 μM G-actin and millimolars of ATP (*40*, *47*). These concentrations are far above the ranges where G-actin and ATP are limiting factors for branch renucleation (Figs. 4, D and E, and 6A). We thus expect that branch dissociation events would nearly all be followed by branch renucleation events, unless they were triggered by a regulatory protein like GMF (Fig. 6B). The fact that GMF and mechanical forces both accelerate debranching but have a very different impact on branch renucleation leads us to propose that the fate of the Arp2/3 complex largely depends on what factor triggers debranching (Fig. 7B).

If debranching is caused by the regulatory protein GMF, it is then very unlikely that a branch will grow back. In this situation, GMF would control the evolution of the branched network toward a more linear architecture, as observed in the rear of lamellipodia or when stress fibers emerge from the cell cortex (*48*, *49*). A fully effective debranching, where no new branch is renucleated, appears essential to fully remodel or disassemble the filament network. As such, we propose that the prime role of GMF in cells may be to prevent branches from growing back, redefining its importance for the control of network architecture and disassembly. Whether and how other proteins affecting branch stability, such as coronin, ADF/cofilin, or cortactin, would affect branch renucleation is now an open question. Other factors should also be investigated, such as the isoform composition of the Arp2/3 complex, which was shown to affect branch stability and its vulnerability to oxidation (*33*, *34*).

In addition, our results on the weakness of the Arp2/3 complex–daughter interface in branch junctions indicate that the pointed end of the dissociated branch is unlikely to be capped by the Arp2/3 complex. We thus propose that the pointed end of dissociated branches may depolymerize or reanneal with barbed ends, thereby contributing to the reorganization of the network.

In contrast to GMF-induced debranching, if the branch dissociates because of mechanical forces applied to the branch junction, then a new branch is likely to grow back. This is a frequent situation in cells, as branched networks can be exposed to substantial mechanical stress. In a lamellipodium, for example, global compression results in forces throughout the entangled filament network, and these forces could have a variety of orientations at the scale of individual branch junctions (*1*). Forces oriented along the axis of global compression would apply a mechanical load on branch junctions similar to the high-angle tension we applied in Fig. 2. In a tensed cortex, the combined action of myosin motors and filament crosslinkers could pull on branch junctions in all directions. Since the orientation of the force has a mild impact on the outcome (Fig. 2), branch renucleation could occur in a broad range of situations.

The forces we have applied in our experiments, in the piconewton range, are comparable to what can be expected for single filaments in cells. Moreover, since debranching is greatly accelerated by force, an ADP-Arp2/3 branch exposed to an increasing load is likely to dissociate rapidly, when the force is still moderate. It will then leave behind an Arp2/3 complex that will rapidly be “rejuvenated” by ATP and from which a new branch will grow. The network would thus rapidly shed old, fragile branches and replace them with new, more resistant branches. This is a new mechanism by which branched actin networks could adapt to mechanical stress by self-repairing, reorganizing, and strengthening. In addition, the renucleated branches are short, allowing them to potentially generate pushing forces without buckling as they elongate. Branch renucleation events can take place away from NPF-decorated surfaces. In the context of the lamellipodium, this could explain why growing barbed ends can sometimes be observed away from the leading edge (*50*–*52*).

Branched actin networks are found in a variety of cellular contexts, with different turnover rates and different functional requirements. Future studies should uncover how the rejuvenation mechanism endowed by branch renucleation contributes to their maintenance and turnover.

## MATERIALS AND METHODS

### Buffers and proteins

#### Proteins

Skeletal muscle actin (UniProt P68135) was purified from rabbit muscle acetone powder following the protocol from (*53*) as described in (*39*). Recombinant cytoplasmic γ-actin (UniProt P63261) was expressed in *Pichia pastoris*, with N-terminal acetylation and His^73^ methylation, following the protocol described in (*54*). The *P. pastoris* strain, expressing both NAA80 acetyltransferase and SETD3 methyltransferase, was a gift from the Balasubramaniam laboratory.

Spectrin-actin seeds from human red blood cells were purified as described in (*39*). The Arp2/3 complex was purified from sheep brain, as previously described in (*55*). For assays using the fluorescently labeled Arp2/3 complex (Fig. 5), we used the recombinant Arp2/3 complex produced in Sf9 insect cells, produced and purified as detailed in (*28*). The Arp2/3 complex from *Saccharomyces cerevisiae* was a gift from A. Michelot.

Recombinant human profilin1 (UniProt P07737) was expressed and purified as described in (*56*). Recombinant N-terminal glutathione *S*-transferase (GST) tag human N-WASP-VCA (amino acids 392 to 505, UniProt O00401) and human WASP-VCA (amino acids 418 to 502, UniProt P42768) were expressed and purified as described in (*29*). Recombinant C-terminal His-tagged drosophila GMF (full length, amino acids 1 to 138, UniProt Q9VJL6, a gift from the Lappalainen laboratory) was expressed and purified as described in (*29*).

#### Protein labeling

Actin was fluorescently labeled on the surface lysine-328, using Alexa Fluor 488, Alexa Fluor 568, or Alexa Fluor 647 NHS ester (Thermo Fisher Scientific), or ATTO 643 NHS ester (Atto-tec) as described in detail in (*57*). The Arp2/3 complex was fluorescently labeled using Alexa Fluor 488 or Alexa Fluor 568 C_5_-maleimide (Thermo Fisher Scientific). The protein solution was prepared for labeling by performing a buffer exchange to remove dithiothreitol (DTT) from the solution. This was accomplished by passing the protein solution through a MicroBiospin 6 column (Bio-Rad). The exchange buffer contained 20 mM Hepes at pH 7.2, 0.2 mM MgCl_2_, and 0.2 mM ATP. The buffer exchange was performed by centrifuging the sample at 1000*g* for 4 min. Next, a 10-fold excess of Alexa Fluor 488 (or Alexa Fluor 568) C_5_-maleimide dissolved in dimethyl sulfoxide (DMSO) was added to the Arp2/3 complex solution and incubated on ice for 1 hour. The reaction was stopped by adding 1 mM DTT to the solution. To remove any unreacted excess dye, a MicroBiospin 6 column (Bio-Rad) was used by centrifugation at 1000*g* for 4 min. We obtained on average 3.5 Alexa dyes per the Arp2/3 complex.

#### Buffer

All microfluidics experiments were performed in F-buffer containing 5 mM tris-HCl (pH 7.0), 50 mM KCl, 1 mM MgCl_2_, 0.2 mM EGTA, 0.2 mM ATP, 10 mM DTT, 1 mM DABCO, supplemented with 0.1% bovine serum albumin (BSA).

### Experiments

#### Data acquisition

Observations were made using a Nikon TiE inverted microscope equipped with a 60× oil-immersion objective and an Evolve 512 EMCCD camera (Photometrics). The temperature of the microfluidics chamber was maintained at 25°C (± 0.2°C) using a collar objective heater (Okolab). We used an azimuthal total internal reflection fluorescence (TIRF) illumination setup (iLAS2, Gataca-systems), with 488-, 561-, and 642-nm tunable lasers (maximum power 100 mW each). The manipulation and adjustment of the TIRF setup were carried out using Metamorph software (version 7.10.4.407). Image analysis was performed using Fiji software (*58*).

#### Basic microfluidics experiment

Microfluidics experiments (Fig. 1) were conducted using polydimethylsiloxane (PDMS; Sylgard) chambers based on the original protocol from (*36*), described in detail in (*59*). Spectrin-actin seeds were flowed in the microfluidics chamber at 20 pM for 2 min. Next, the surface was passivated by exposing it to a solution containing 5% BSA for 10 min. After passivation, surface-anchored mother filaments were polymerized by flowing in 0.6 μM 10% Alexa Fluor 488–labeled G-actin for 5 to 10 min. Next, to induce the nucleation of actin filament branches at a sufficient density, mother filaments were exposed to a solution of 20 nM Arp2/3 complex, 50 nM VCA, and 0.4 μM 10% Alexa Fluor 568–labeled G-actin for 45 s to 2 min. However, it should be noted that, in all experiments, the indicated time of exposure to the branching solution refers to the period following the increase of the flow rate, after the reservoir containing the protein solution is plugged to the microfluidic device. During this time, mother filaments were exposed to a protein concentration that progressively increased from zero to 70% (after 45 s) to 100% (after 2 min) of the nominal concentration (*59*). Afterward, actin filament branches and mother filaments were aged with a low flow of 0.3 or 1 μM 10% Alexa Fluor 568–labeled G-actin for 20 min. Finally, they were exposed to debranching conditions: 0.3 μM 10% Alexa Fluor 568–labeled G-actin with different flow rates (to apply different forces). The dissociation of branch filaments and their renucleation were observed over time and acquired with the acquisition rate of 1 frame every 10 s.

#### Experiment on force orientation

In our experiment on force orientation (Fig. 2), spectrin-actin seeds were flowed to the microfluidics chamber at 20 pM for 2 min. Then, the surface was rinsed by buffer and passivated by 0.5% BSA and 0.1% biotin-BSA for 10 to 15 min. Surface-anchored mother filaments were polymerized in three steps sequentially to create three segments: Starting from the seed, first a non–biotin-actin segment (by 0.8 μM 10% Alexa Fluor 488–labeled G-actin), then a biotin-actin segment (by 0.8 μM 6% Alexa Fluor 488–labeled G-actin, 50% to 75% biotin labeled), and finally a short non-biotin actin segment (by 0.8 μM 10% Alexa Fluor 488–labeled G-actin). Next, the filaments were exposed to 20 to 50 nM neutravidin for 2 min. Afterward, mother filaments were exposed to 20 nM Arp2/3 complex, 50 nM VCA, and 0.4 μM 10% Alexa Fluor 568–labeled G-actin for 45 s to 2 min to nucleate actin filament branches at sufficient density. Then, the actin filament branches, as well as mother filaments, were elongated with a low flow of 0.3 μM 10% Alexa Fluor 568–labeled G-actin for 4 min. Finally, they were exposed to debranching conditions: 0.3 μM 10% Alexa Fluor 568–labeled G-actin, flowing perpendicular to the mother filament orientation (from left-hand side channel to all three other channels). The dissociation of branch filaments and their renucleation were recorded over time at 1 frame every 10 s. For each branch, the angle between the branch (local direction of the flow) and the mother filament at the branch junction was measured manually using the Fiji software.

#### Experiment on unlabeled cytoplasmic γ-actin

In our experiment on unlabeled cytoplasmic γ-actin (Fig. 3C), spectrin-actin seeds were flowed into the microfluidics chamber at 20 pM for 2 min. Then, the surface was rinsed with F-buffer and passivated by being exposed to a solution containing 5% BSA for 10 min. After rinsing, surface-anchored mother filaments were polymerized from spectrin-actin seeds in three steps sequentially to create three segments: starting from the seed, first a labeled α-actin segment (by 0.6 μM 10% Alexa Fluor 488–labeled α G-actin), then an unlabeled γ-actin segment (by 0.6 μM unlabeled γ G-actin), and then again a labeled α-actin segment (by 0.6 μM 10% Alexa Fluor 488–labeled α G-actin). Next, the mother filaments were exposed to 20 nM Arp2/3 complex, 50 nM VCA, and 0.4 μM unlabeled γ G-actin for 1 min. Afterward, the filaments and branched junctions were aged while exposed to a solution of 0.3 μM 10% Alexa Fluor 568–labeled α G-actin, circulating at a low flow rate for 20 min. Finally, they were exposed to 0.3 μM 10% Alexa Fluor 568–labeled G-actin at a flow rate that applied a 1- to 1.5-pN force to the branch junction. For comparison, we monitored over time (acquisition rate of 1 frame every 10 s) the debranching and renucleation of branches that were nucleated from the side of mother filaments, either along labeled α-actin or unlabeled γ-actin segments.

#### Experiment on profilin-actin

In our experiment on profilin-actin (Fig. 3D), spectrin-actin seeds were flowed into the microfluidics chamber at 20 pM for 2 min. Then, the surface was rinsed with F-buffer and passivated by being exposed to a solution containing 5% BSA for 10 min. After rinsing, surface-anchored mother filaments were polymerized by 0.6 μM 10% Alexa Fluor 488–labeled G-actin. Next, mother filaments were exposed to 20 nM Arp2/3 complex, 50 nM VCA, and 0.4 μM 10% Alexa Fluor 568–labeled G-actin for 1 min to nucleate actin filament branches. In the next step, two experimental solutions (actin alone or equimolar amounts of actin and profilin) were introduced into the microfluidics chamber, side by side at equal flow rates, resulting in each solution occupying half of the chamber. By alternating stage positions during the acquisitions of images, this experimental setup allowed us to simultaneously investigate two distinct conditions within the same chamber while keeping all other variables constant. The dissociation of branch filaments and their renucleation, for each debranching condition, were observed over time and acquired with the acquisition rate of 1 frame every 10 s.

#### Experiment on comparison of the first and second generation

In our experiment on comparison of the first and second generation (Fig. 4, A and B), the spectrin-actin seeds were introduced into the microfluidics chamber at a concentration of 20 pM and allowed to flow for 2 min. Subsequently, the surface was rinsed with F-buffer and passivated by being exposed to a solution containing 5% BSA for a duration of 10 min. After rinsing, surface-anchored mother filaments were polymerized by 0.8 μM 10% Alexa Fluor 488–labeled G-actin. Next, mother filaments were exposed to 20 nM Arp2/3 complex, 50 nM VCA, and 0.4 μM 10% Alexa Fluor 568–labeled G-actin for 45 s to nucleate actin filament branches. Then, branches and mother filament were exposed to 0.4 μM 10% Alexa Fluor 568–labeled G-actin for 90 s to elongate nucleated branches. Afterward, branches were aged for 20 min with a low flow of 0.18 μM 10% Alexa Fluor 568–labeled G-actin. Finally, branches were exposed to debranching conditions: 0.3 μM 10% Alexa Fluor 568–labeled G-actin. The dissociation of branch filaments, renucleation of second-generation branches, and their dissociation were observed over time and acquired with the acquisition rate of 1 frame every 10 s.

#### Experiment on ATP depletion by resin treatment

In our experiment on ATP depletion by resin treatment (Fig. 4D), ATP in the solution of actin in F-buffer was removed by two consecutive treatments (2 × 15 min) with 5% (w/w) Ion Exchange Resins (AG 1-X8 Resin, Bio-Rad).

Spectrin-actin seeds were flowed to the microfluidics chamber at 20 pM for 2 min. Then, the surface was rinsed with F-buffer and passivated by exposing it to a solution containing 5% BSA for 10 min. After rinsing, surface-anchored mother filaments were polymerized by 0.7 μM 10% Alexa Fluor 488–labeled G-actin. Then, mother filaments were exposed to 20 nM Arp2/3 complex, 50 nM VCA, and 0.4 μM 10% Alexa Fluor 568–labeled G-actin for 1 min to nucleate actin filament branches. Next, two experimental solutions were flowed side by side to the microfluidics chamber at equal flow rates so that each of them only occupied half of the chamber. Experimental solutions were resin-treated solution (1 μM 10% Alexa Fluor 568–labeled G-actin, [ATP] < 1 μM), rescued resin-treated solution of actin (1 μM 10% Alexa Fluor 568–labeled G-actin, ATP added after treatment), and control (1 μM 10% Alexa Fluor 568–labeled G-actin, [ATP] = 200 μM). The dissociation of branch filaments and their renucleation, for each debranching condition, were observed over time and acquired with the acquisition rate of 1 frame every 10 s.

#### Experiment on labeled Arp2/3 complex

In our experiment on labeled Arp2/3 complent (Fig. 5), the chamber was incubated with a solution of poly-L-lysine-polyethylene glycol (PLL-PEG) (20 mg/ml) in phosphate-buffered saline (PBS) for 90 min. Next, it was incubated with 100 pM spectrin-actin seeds for 2 min. To enhance passivation, the chamber was further incubated with a solution containing 1% BSA and casein (0.5 mg/ml) for 30 more minutes. After rinsing, surface-anchored mother filaments were polymerized by 0.7 μM 10% Alexa Fluor 647–labeled G-actin. Then, mother filaments were exposed to 20 nM Alexa Fluor 568 (or 488)–labeled Arp2/3 complex, 50 nM VCA, and 0.4 μM 10% Alexa Fluor 488 (or 568)–labeled G-actin for 45 s to nucleate actin filament branches. Filaments were aged with 0.3 μM 10% Alexa Fluor 488 (or 568)–labeled G-actin for 30 min. Finally, filaments were exposed to a high flow of F-buffer containing either 200 μM ATP or 200 μM ADP. The dissociation of branch filaments and departure of labeled Arp2/3 complexes were observed over time (at 50% laser power) and recorded with an acquisition rate of 1 frame every 0.8, 1.5, or 5 s in the presence of ATP, or 1 frame per second in the presence of ADP.

#### Experiment on actin concentration versus renucleation ratio

In our experiment on actin concentration versus renucleation ratio (Fig. 6A), spectrin-actin seeds were flowed into the microfluidics chamber at 20 pM for 2 min. The surface was then rinsed with buffer and passivated for 10 min using 5% BSA. Following this, mother filaments anchored to the surface were polymerized using 0.8 μM 10% Alexa Fluor 488–labeled G-actin. Mother filaments were exposed to 20 nM Arp2/3 complex, 50 nM VCA, and 0.4 μM 10% Alexa Fluor 568–labeled G-actin for 45 s to 1 min to nucleate actin filament branches. Next, mother filament and branches were elongated and aged with a low concentration of actin (0.3 or 0.4 μM 10% Alexa Fluor 568–labeled G-actin) for 30 min. Finally, they were exposed to debranching conditions: different concentrations of 10% Alexa Fluor 568–labeled G-actin. The average force for each experiment was calculated by determining the average length of branches and the total flow rate in the chamber. Flow rates were adjusted to maintain an average force of 1 to 2 pN. The dissociation of branches and their subsequent renucleation were observed over time and recorded at an acquisition rate of 1 frame per 10 s.

To control the length of actin branches during the experiments performed with high actin concentrations (>1.2 μM), filament and branches were exposed to 1.5 μM capping protein (CP) for 60 s and allowed to age further up to 30 min while remaining capped (using 0.3 μM 10% Alexa Fluor 568–labeled G-actin), before being exposed to the debranching conditions mentioned above.

#### Experiment on the impact of GMF on debranching and renucleation

In our experiment on the impact of GMF on debranching and renucleation (Fig. 7), spectrin-actin seeds were flowed into the microfluidics chamber at 20 pM for 2 min. Then, the surface was rinsed by buffer and passivated by 5% BSA for 10 min. After rinsing, surface-anchored mother filaments were polymerized by 0.8 μM 10% Alexa Fluor 488–labeled G-actin. Mother filaments were exposed to 20 nM Arp2/3 complex, 50 nM VCA, and 0.4 μM 10% Alexa Fluor 568–labeled G-actin for 1 min to nucleate actin filament branches. Next, mother filament and branches were aged with 0.4 μM 10% Alexa Fluor 568–labeled G-actin for 30 min. Finally, the chamber was exposed to the debranching conditions: different concentrations of GMF (up to 1500 nM) and 0.3 (and 0.6) μM 10% Alexa Fluor 568–labeled G-actin. The dissociation of branch filaments and renucleation of second-generation branches were observed over time and acquired with the acquisition rate of 1 frame every 10 s.

#### Experiment with a bright fluorescent pointed end region of branches

In our experiment with a bright fluorescent pointed end region of branches (fig. S3, B to D), spectrin-actin seeds were flowed into the microfluidics chamber at a concentration of 20 pM for 2 min. The chamber surface was then rinsed with buffer and passivated with 5% BSA for 10 min. Before performing the experiment, we performed a speckle experiment.

##### Speckle experiment

To determine the fluorescence intensity of a single Alexa Fluor 488 fluorophore on actin, we monitored filaments sparsely labeled with Alexa Fluor 488. Mother filaments were polymerized using 0.8 μM labeled G-actin (containing 10% Alexa Fluor 568–actin and 0.1% Alexa Fluor 488–actin). Then, by testing different laser powers and acquisition times, we determined a set of illumination conditions (100% laser power, 1000 ms acquisition time) that ensure the clear detection of a single Alexa Fluor 488 fluorophore in our setup.

##### Main experiment

Starting from a fresh coverslip surface, mother filaments were polymerized using 0.6 μM 10% ATTO 643–labeled G-actin from spectrin-actin seeds. To nucleate actin filament branches, the mother filaments were exposed to 20 nM Arp2/3 complex, 50 nM VCA, and 0.4 μM 46.8% Alexa Fluor 488–labeled G-actin for 45 s. Highly labeled branch junctions were observed with an acquisition rate of 1 frame every 5 s. The filaments and branch junctions were aged for 20 min in the presence of 0.2 μM 10% Alexa Fluor 568–labeled G-actin, followed by exposure to debranching conditions using 0.5 μM 10% Alexa Fluor 568–labeled G-actin for 10 min. Debranching and renucleation events were observed and acquired with an acquisition rate of 1 frame every 20 s. During that time, the Alexa Fluor 488 fluorophores were not excited to avoid photobleaching (the 488-nm laser was turned off). Finally, to assess the presence of Alexa Fluor 488–actin subunits from the first generation of branches at the junction of renucleated branches, filaments were monitored at an acquisition rate of 1 frame every 5 s, using all three different wavelengths (488-, 561-, and 642-nm excitation lasers) and using the illumination conditions determined in the speckle experiment described above to ensure that single Alexa Fluor 488 molecules would be detected.

### Data analysis

#### Branch survival functions

The fraction of surviving branches as a function of time is computed by the Kaplan-Meier method using GraphPad Prism version 9 for Windows. The error bars represent SE calculated by the method of Greenwood.

#### Branch renucleation ratio

The branch renucleation ratio represents the ratio of the number of renucleated branches to the number of dissociated branches. Error bars show the binomial SD.

#### Calculation of the force applied to the branch junction

The tensile force exerted on branches is a result of the friction (viscous drag) of the fluid flow applied to the filaments. As characterized previously in (*37*), the pulling force on the branch junction is the product of the length of the branch, the local flow velocity, and the longitudinal friction coefficient per unit length of the actin filament (η_actin_ = 6 × 10^−4^ pN μm^−2^ s). In each experiment, the average force and SD of the force were determined for the population of analyzed branches.

#### Off-rate of Arp2/3 complex in the presence of ADP or ATP

The off-rate of the lone Arp2/3 complex (in the presence of ADP or ATP) (Fig. 5, C and D) was derived from the exponential fit of the survival fraction of the Arp2/3 complex on mother filament over time, with a 95% confidence interval, using GraphPad Prism version 9 for Windows.

#### On-rate of actin monomers

The branch renucleation ratio can be viewed as the probability to renucleate a branch after the initial branch has dissociated. In the frame of our model (Fig. 6B), this can be written as the product of three terms:

(1) the probability for the Arp2/3 complex to stay on the mother filament as the branch dissociates;

(2) the probability for the surviving ADP–Arp2/3 complex to bind ATP before dissociating from the mother filament;

(3) the probability for the ATP–Arp2/3 complex to bind actin monomers before dissociating from the mother filament.

Term (1) depends on the force *F* applied to the branch. In the following, we call it *A*(*F*). Term (2) depends on the ATP concentration. On the basis of our results (summarized in Fig. 6B), the surviving ADP–Arp2/3 complex dissociates from the mother filament at a rate equal to 0.66 s^−1^ and binds ATP with a rate constant of >6 μM^−1^ s^−1^; thus, a lower estimate of term (2) is $\frac{6\left[\text{ATP}\right]}{6\left[\text{ATP}\right]+0.66}$ , which equals 0.999 with 200 μM ATP. We thus consider that the dissociation of the ADP–Arp2/3 complex from the mother filament, before reloading ATP, is negligible in the condition of our experiments done with 200 μM ATP (as in Fig. 6A).

Term (3) depends on the concentration of actin monomers, *C*. It can be written as $\frac{k_{\text{on}}\left(C-C_{C}\right)}{k_{\text{on}}\left(C-C_{C}\right)+k_{\text{off}}^{\text{ATP}-\text{Arp}2/3}}$ , where $k_{\text{off}}^{\text{ATP}-\text{Arp}2/3}$ is the detachment rate of the ATP–Arp2/3 complex from the side of the mother filament (0.57 s^−1^; Fig. 5D), *k*_on_ is the on-rate constant for G-actin binding to the ATP–Arp2/3 complex on the mother filament, and *C*_C_ is the actin critical concentration for that reaction.

Thus, in conditions where there is enough ATP for term (2) to be approximated to 1, we can write:

$$\text{branch renucleation ratio}=\frac{A\left(F\right)\times k_{\text{on}}\left(C-C_{C}\right)}{k_{\text{on}}\left(C-C_{C}\right)+k_{\text{off}}^{\text{ATP}-\text{Arp}2/3}}$$

This is the formula used to fit the data in Fig. 6A, with *A*(*F*), *C*_C_, and *k*_on_ as free parameters. *A*(*F*) is considered constant because the data in Fig. 6A were acquired at similar forces (1.54 ± 0.23 pN).

#### Relative strength of bonds between Arp2/3 complex and filaments

To interpret our results in terms of the relative strengths of the bonds between the Arp2/3 complex and the mother and daughter filaments, we propose the following simple calculations. Note that, since the flow applies negligible forces to the surviving Arp2/3 complex and to the first actin subunits that bind to it, we expect that only the branch dissociation step can be force-sensitive.

We consider that each interface has its own force-dependent dissociation rate: the mother–Arp2/3 complex bond ruptures with rate $k_{\text{off}}^{\text{mother}}\left(F\right)$, and the Arp2/3 complex–daughter bond ruptures with rate $k_{\text{off}}^{\text{daughter}}\left(F\right)$ , where *F* is the pulling force applied to the branch.

The branch thus dissociates from the mother with rate $k_{\text{off}}^{\text{mother}}\left(F\right)+k_{\text{off}}^{\text{daughter}}\left(F\right)$ . The probability for Arp2/3 to remain on the mother corresponds to the probability that the Arp2/3-daughter interface ruptures first, which is equal to $\frac{k_{\text{off}}^{\text{daughter}}\left(F\right)}{k_{\text{off}}^{\text{mother}}\left(F\right)+k_{\text{off}}^{\text{daughter}}\left(F\right).}$

Since $k_{\text{off}}^{\text{mother}}\left(F\right)+k_{\text{off}}^{\text{daughter}}\left(F\right)$ strongly increases with *F* (Fig. 1D), while $\frac{k_{\text{off}}^{\text{daughter}}\left(F\right)}{k_{\text{off}}^{\text{mother}}\left(F\right)+k_{\text{off}}^{\text{daughter}}\left(F\right)}$ moderately decreases with *F* (Fig. 1F), we can conclude that $k_{\text{off}}^{\text{mother}}\left(F\right)$ and $k_{\text{off}}^{\text{daughter}}\left(F\right)$ vary similarly and that both increase with *F*. In other words, both interfaces can be viewed as “slip bonds,” in the force range we have studied.

Since $\frac{k_{\text{off}}^{\text{daughter}}\left(F\right)}{k_{\text{off}}^{\text{mother}}\left(F\right)+k_{\text{off}}^{\text{daughter}}\left(F\right)}$ decreases with increasing force, we can conclude that $k_{\text{off}}^{\text{mother}}\left(F\right)$ increases with *F* more strongly than $k_{\text{off}}^{\text{daughter}}\left(F\right)$ . In other words, the mother–Arp2/3 complex interface appears more sensitive to force than the Arp2/3 complex–daughter interface, in the force range we have studied.

#### GMF-induced branch dissociation rates

The branch dissociation rate in the presence of GMF was derived from the exponential fit of survival fractions (Fig. 7A), with a 95% confidence interval, using GraphPad Prism.

The dependence of the branch dissociation rates as a function of GMF concentration was fitted by the classical Michaelis-Menten kinetics equation, with *k* (dissociation rate without GMF), *k*_max_ (maximum dissociation rate), and *K*_D_ (dissociation constant of GMF on the branch junction) as free parameters:

$$\text{branch dissociation rate}=k+\frac{\left[\text{GMF}\right]}{\left[\text{GMF}\right]+K_{D}}\left(k_{\text{max}}-k\right)$$

#### Fluorescent intensity of renucleated branch at the junction over time

The intensity of each individual branch junction was measured as the average intensity in a 4 × 4–pixel region over time at each time interval (fig. S2). The background intensity in the vicinity of the same branch junction was subtracted from the measured intensity. For each branch, the intensity reaches a plateau when the branch grows out of the 4 × 4–pixel region, and this plateau is used to normalize the fluorescent signal (fig. S2B). The plots in fig. S2B show, for each experiment, the normalized intensity over time, averaged over the observed population of renucleated branches.
