# Supplementary material for: Regeneration of actin filament branches from the same Arp2/3 complex
Source: Sci Adv. 2024 Jan 26;10(4):eadj7681. doi: 10.1126/sciadv.adj7681 (PMC10816697; doi:10.1126/sciadv.adj7681)
Supplement: Supplementary file 1 — Figs. S1 to S6 Legend for data file S1 [file sciadv.adj7681_sm.pdf]

Science Advances
AAAS

Supplementary Materials for

Regeneration of actin filament branches from the same Arp2/3 complex

Foad Ghasemi *et al.*

Corresponding author: Antoine Jégou, antoine.jegou@ijm.fr; Guillaume Romet-Lemonne, romet@ijm.fr

Sci. Adv. **10**, eadj7681 (2024)
DOI: 10.1126/sciadv.adj7681

The PDF file includes:

Figs. S1 to S6
Legend for data file S1

Other Supplementary Material for this manuscript includes the following:

Data file S1

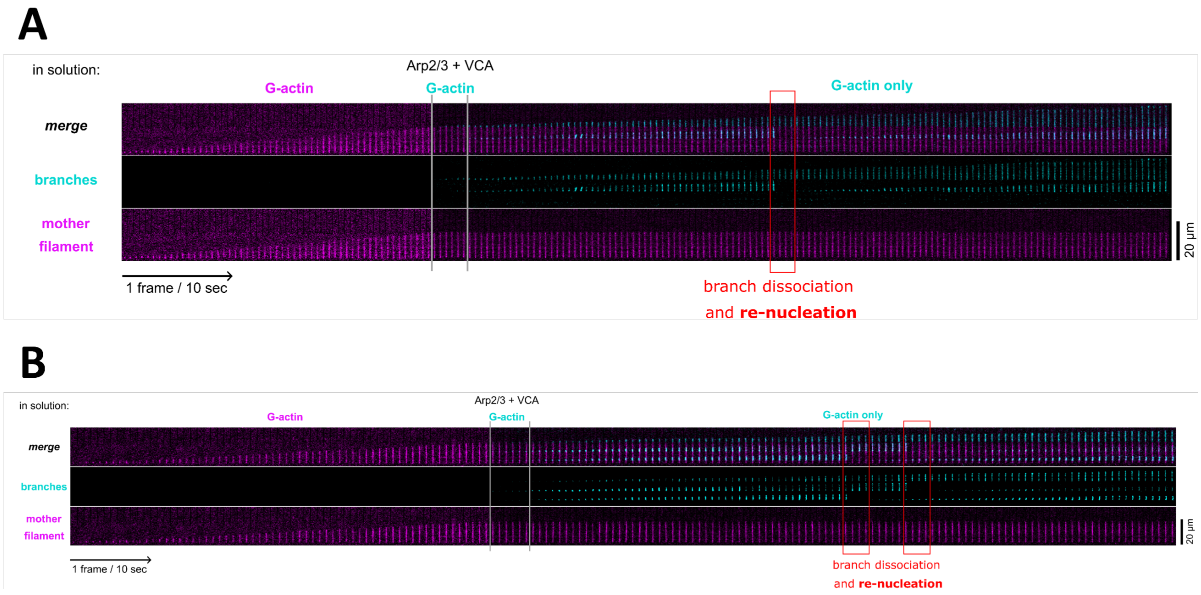

Fig. S1. Additional examples of timelapses showing branch dissociation and re-nucleation, similar to Fig 1C,E, over longer time scales. (**A**) One branch is visible. It dissociates and another one is re-nucleated at the same location. (**B**) Two branches are visible on the same mother filament. Each dissociates and each is replaced by a re-nucleated branch. In (A) and (B), the elongation of the barbed end is clearly visible, during the different steps of the experiment. The red boxes indicate branch dissociation and re-nucleation events.

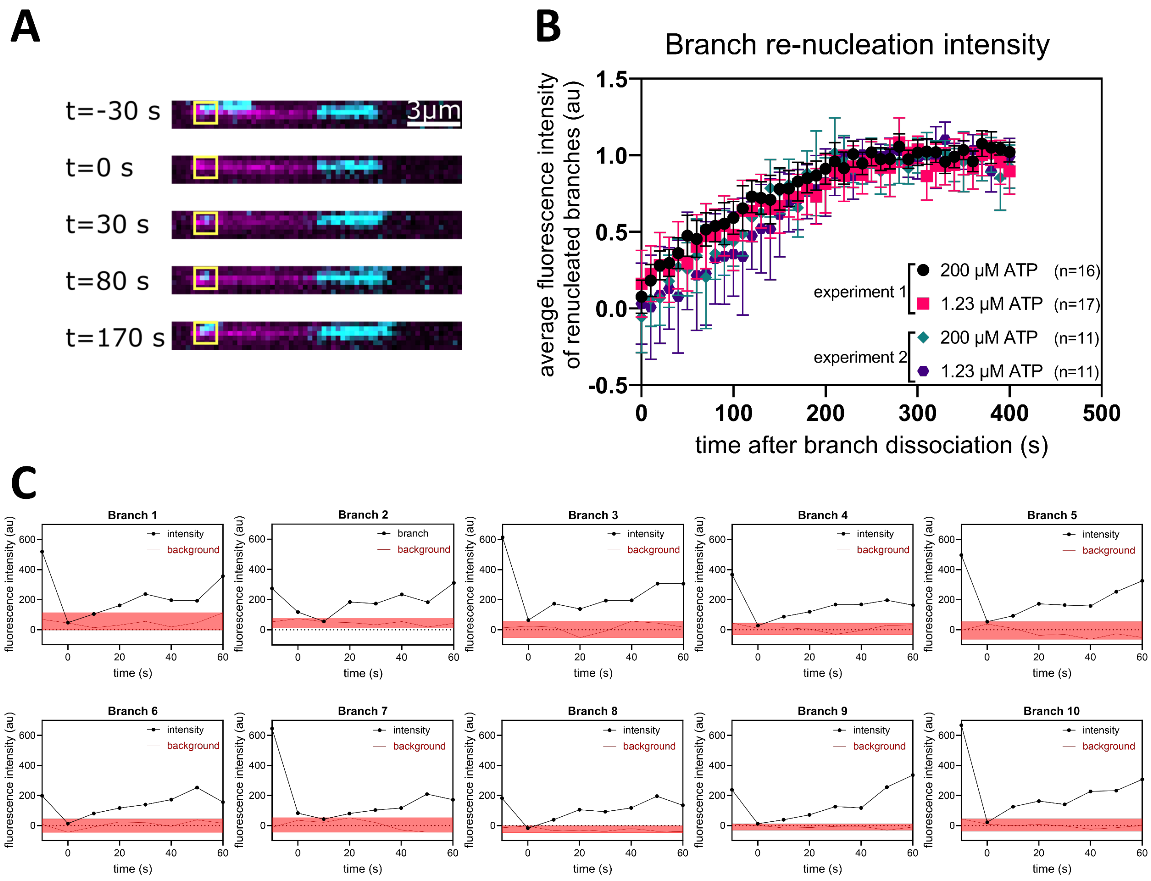

Fig. S2. Branch re-nucleation is observed immediately following branch dissociation.

(**A**) Time-lapse of branch dissociation followed by branch re-nucleation. The average intensity of the branch junction was measured within a 4x4-pixel region, over time. Mother filaments and branches were polymerized with actin labeled by Alexa-488 and Alexa-568, respectively. (**B**) Average fluorescence intensity of n re-nucleated branches over time, normalized to the maximum signal for each branch. Time zero marks branch dissociation, determined as the frame when the branch (Alexa-568) signal is lost. No lag was detected between branch dissociation and branch re-nucleation.

(**C**) Same measurement as in B, with a stronger illumination (laser power twice as strong), and showing the variation in fluorescence intensity for 10 individual branches. The intensity is not normalized. Time zero is defined by the drop of fluorescence intensity (branch dissociation). The red shaded region indicates the amplitude of the fluctuations in the background intensity measured a few pixels away from the filament.

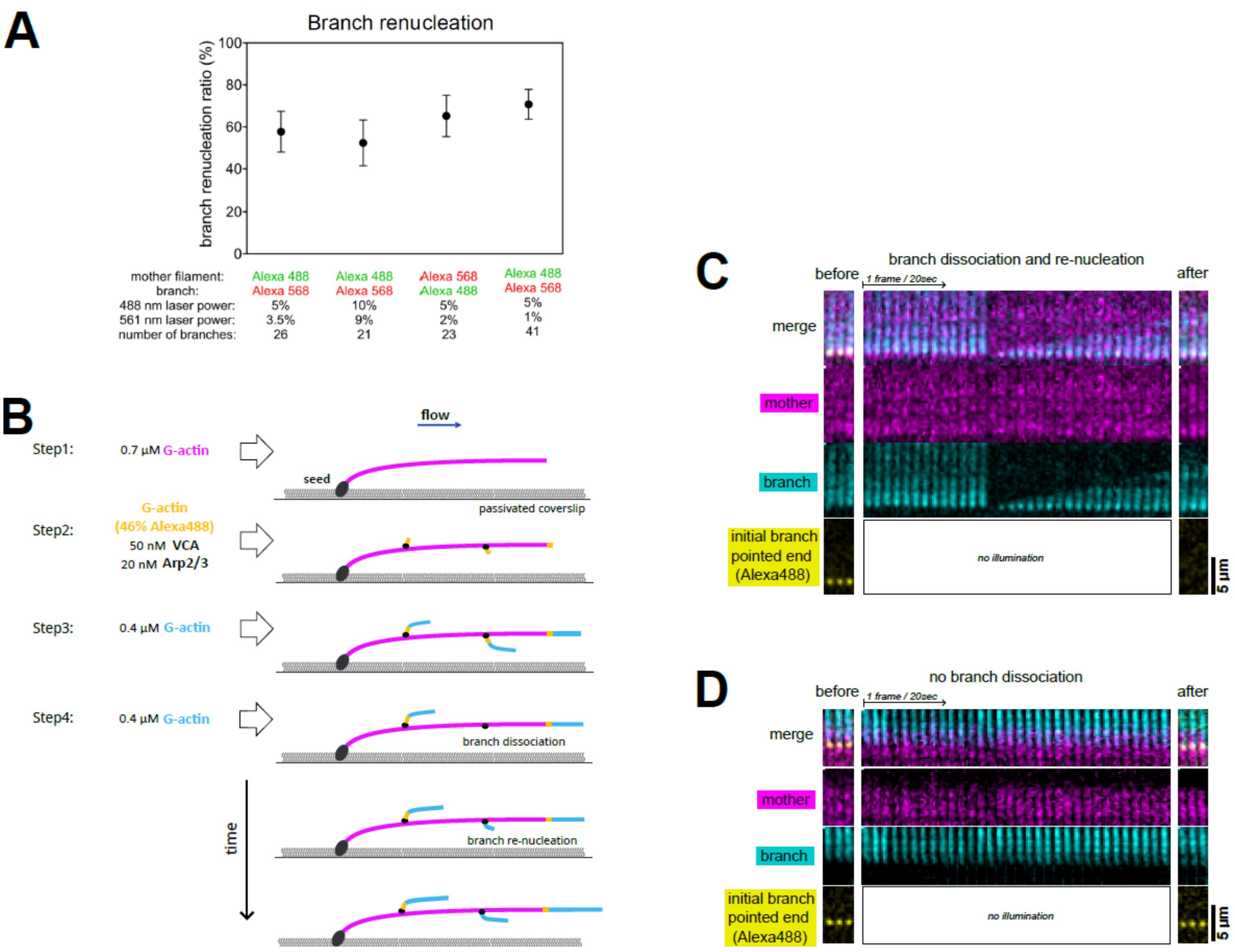

Fig. S3. Branch dissociation events are not due to severing of the branch close to the branch junction. (**A**) Effect of laser power and labeling on branch re-nucleation. (**B**) Schematic of experiment designed to look at bright Alexa-488-labeled segments of branches close to the branch junction. Branches with a clearly visible Alexa-488 signal at their branch junction after the nucleation of the initial branch (step 3) were further monitored over time and analyzed (step 4). (**C**) Time lapse of a branch that dissociated and re-nucleated (during step 4, as shown in (B)), shown as an example: after re-nucleation, the Alexa-488 signal is no longer detectable, in conditions where a single fluorophore would be detected. Nearly none of these re-nucleated branches (1 out of 16) had a detectable Alexa-488 signal at their branch junction at the end of the experiment. (**D**) Time lapse of a branch that did not dissociate (during step 4, as shown in (B)), shown as an example: the Alexa-488 signal is still visible at the end of the experiment. These branches provide an internal control. Nearly all of them (11 out of 12) still had a detectable Alexa-488 signal at the end of the experiment.

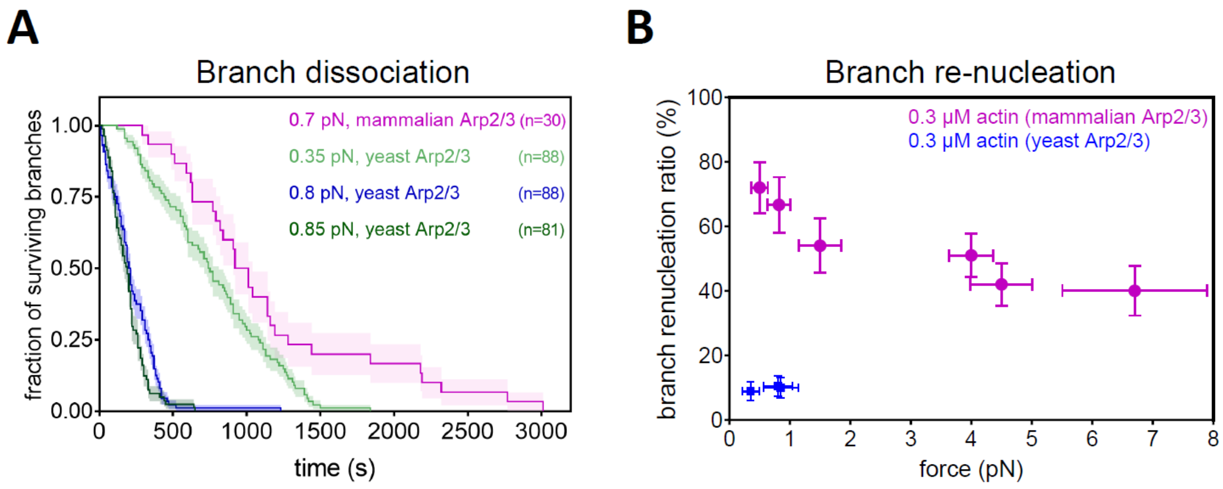

Fig. S4. Branches nucleated by Arp2/3 complex from S. Cerevisiae dissociate faster and barely re-nucleate. (**A**) Comparison of the dissociation of branches nucleated from mammalian Arp2/3 complex (pink curve) and *S. Cerevisiae* Arp2/3 complex (other curves), exposed to different pulling forces. Each curve is from a single experiment, where the branches were pre-aged for 4 minutes (before t=0). In all experiments, mammalian alpha-skeletal actin was used. (**B**) Comparison of the ratio of re-nucleated branches, from mammalian Arp2/3 complex (pink) and *S. Cerevisiae* Arp2/3 complex (blue), for different pulling forces. The data for mammalian Arp2/3 complex is the same as in Fig 1F. For yeast Arp2/3 complex, each point is from a single experiment, monitoring, from left to right, n=90, 95, 95 branches. In all experiments, mammalian alpha-skeletal actin was used.

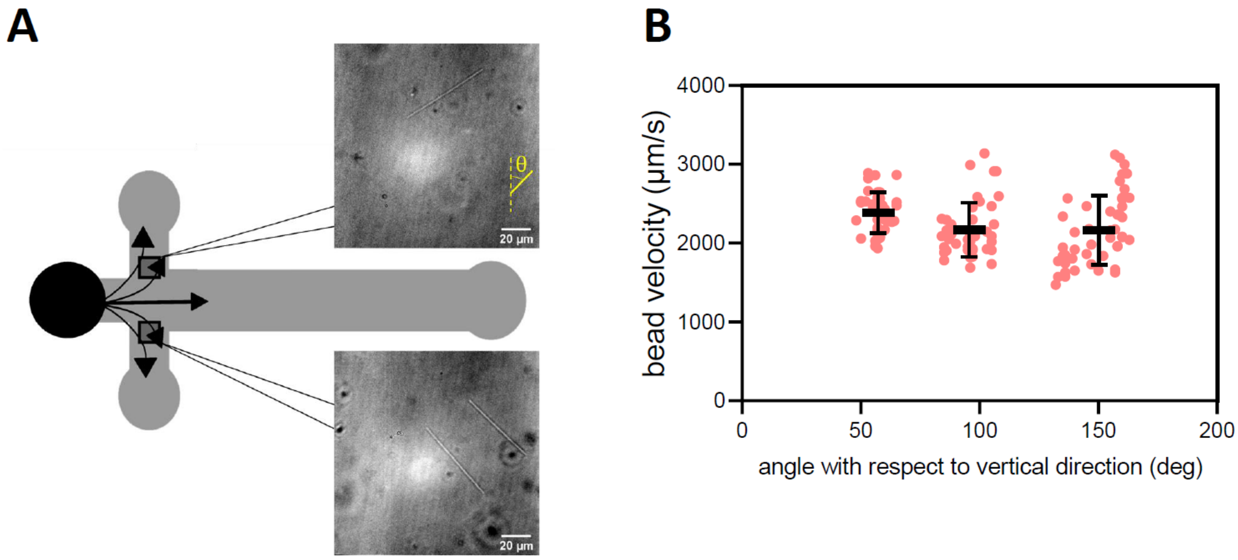

Fig. S5. Flow velocity as a function of flow direction. (**A**) The microscope images (transmitted light) show the traces of one bead (top) and 2 beads (bottom) passing in two different regions of the microchamber. The images were acquired over 30 ms and beads have a diameter of 3 μm. (**B**). Each data point indicates the velocity of a single bead, traveling in a direction making an angle θ with respect to the vertical direction on the images. The black marks indicate the average velocities and standard deviations for 3 subpopulations of n=39, 43, 51 beads (from left to right) with similar angles.

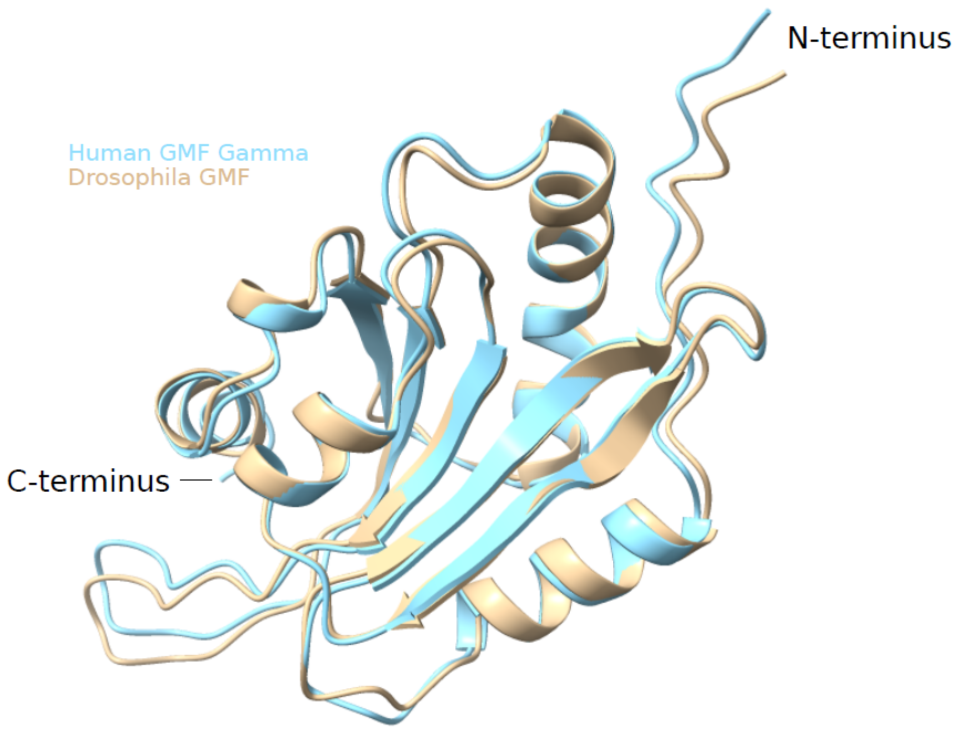


```
sp|O60234|GMFG_HUMAN      MSDSLVVCEVDPELTEKLRKFRFRKETDNAAIIMKVDKDRQMVVLEEEFQNISPEELKME 60
sp|Q9VJL6|GMF_DROME       -MSDNQICDISNEVLEELKKFRFSKSKNNAALILKVDREKQTVVLDEFIDDISVDELQDT 59
                            ..  :*::. *: *:*:**** *..:***:*:***:::* ***:* :::** :**:

sp|O60234|GMFG_HUMAN      LPERQPRFVVYSYKYVHDDGRVSYPLCFIFSSPVGCKPEQQMMYAGSKNRLVQTAELTKV 120
sp|Q9VJL6|GMF_DROME       LPGHQPRYVIYTYKMVHDDQRISYPMCFIFYTPRDSQIELQMMYACTKSALQREVDLTRV 119
                          ** :***:*:*:** **** *:***:**** :* ..: * ***** :*. * : .:**:*

sp|O60234|GMFG_HUMAN      FEIRTTDDLTEAWLQEKLSFFR 142
sp|Q9VJL6|GMF_DROME       YEIRELDELTEEWLKAKLK--- 138
                          :***  *:*** **: **.
```

Fig. S6. Comparing GMF from *drosophila* and *homo sapiens*.
(Top) Aligned Alphafold-generated structures from *Drosophila melanogaster* GMF (Uniprot Q9VJL6, in light brown) and *Homo sapiens* GMF Gamma (Uniprot O60234, in light blue). Alignment has been performed using ChimeraX.
(Bottom) Protein sequence alignment using clustalW. Protein similarity is 81%.

Supplementary data file

Excel spreadsheet containing the data for all the plots shown in the figures of the article. Each figure panel corresponds to a different sheet, indicated by its thumbnail.
